# Automatic Quantification of Cardiomyocyte Dimensions and Connexin 43 Lateralization in Fluorescence Images

**DOI:** 10.3390/biom10091334

**Published:** 2020-09-17

**Authors:** Antoni Oliver-Gelabert, Laura García-Mendívil, José María Vallejo-Gil, Pedro Carlos Fresneda-Roldán, Katarína Andelová, Javier Fañanás-Mastral, Manuel Vázquez-Sancho, Marta Matamala-Adell, Fernando Sorribas-Berjón, Carlos Ballester-Cuenca, Narcisa Tribulova, Laura Ordovás, Emiliano Raúl Diez, Esther Pueyo

**Affiliations:** 1 Biomedical Signal Interpretation and Computational Simulation (BSICoS), Institute of Engineering Research (I3A), University of Zaragoza & Instituto de Investigación Sanitaria (IIS), 50018 Zaragoza, Spain; aoliver@unizar.es (A.O.-G.); lgmendivil@unizar.es (L.G.-M.); lordovas@unizar.es (L.O.); 2 Department of Cardiovascular Surgery, University Hospital Miguel Servet, 50018 Zaragoza, Spain; jmvallejo@salud.aragon.es (J.M.V.-G.); pcfresneda@salud.aragon.es (P.C.F.-R.); jfananas@salud.aragon.es (J.F.-M.); drmvazquez@yahoo.es (M.V.-S.); mmatamala80@hotmail.com (M.M.-A.); juanfernandosorribas@gmail.com (F.S.-B.); cballester@salud.aragon.es (C.B.-C.); 3 Centre of Experimental Medicine, SAS, 84104 Bratislava, Slovakia; katarina.andelova@savba.sk (K.A.); narcisa.tribulova@savba.sk (N.T.); 4 Aragon Agency for Research and Development (ARAID), 50018 Zaragoza, Spain; 5 Institute of Experimental Medicine and Biology of Cuyo (IMBECU), CONICET, 855 5500 Mendoza, Argentina; 6 Biomedical Research Networking Center in Bioengineering, Biomaterials and Nanomedicine (CIBER-BBN), 50018 Zaragoza, Spain

**Keywords:** automated quantification, Connexin 43, fluorescent microscopy, lateralization, cardiomyocytes

## Abstract

Cardiomyocytes’ geometry and connexin 43 (CX43) amount and distribution are structural features that play a pivotal role in electrical conduction. Their quantitative assessment is of high interest in the study of arrhythmias, but it is usually hampered by the lack of automatic tools. In this work, we propose a software algorithm (Myocyte Automatic Retrieval and Tissue Analyzer, MARTA) to automatically detect myocytes from fluorescent microscopy images of cardiac tissue, measure their morphological features and evaluate the expression of CX43 and its degree of lateralization. The proposed software is based on the generation of cell masks, contouring of individual cells, enclosing of cells in minimum area rectangles and splitting of these rectangles into end-to-end and middle compartments to estimate CX43 lateral-to-total ratio. Application to human ventricular tissue images shows that mean differences between automatic and manual methods in terms of cardiomyocyte length and width are below 4 μm. The percentage of lateral CX43 also agrees between automatic and manual evaluation, with the interquartile range approximately covering from 3% to 30% in both cases. MARTA is not limited by fiber orientation and has an optimized speed by using contour filtering, which makes it run hundreds of times faster than a trained expert. Developed for CX43 studies in the left ventricle, MARTA is a flexible tool applicable to morphometric and lateralization studies of other markers in any heart chamber or even skeletal muscle. This open-access software is available online.

## 1. Introduction

The size and shape of cardiomyocytes (CMs) are major determinants of the electrical propagation in the heart [1,2,3,4]. The cellular membrane acts as a capacitor and the capacitance correlates with the total surface of the cell. CMs’ volume modulates the amount of specific intracellular resistance. Conduction through extracellular spaces depends on tissue structure. In particular, the arrangement of myocytes, non-myocyte cells and connective tissue influence the properties of the extracellular conductor [5].

Electrical conduction in the heart is also highly determined by the expression and distribution of connexins. Connexins are the proteins that form intercellular channels in the gap junctions of the myocardial tissue [6]. Gap junctions allow electrical and metabolic connection between CMs, giving the myocardial tissue a syncytium behavior. Gap junctions are concentrated at the intercalated discs between myocytes, located at their longitudinal ends. Under stressful conditions, connexins can misslocalize to the lateral sides of the CMs. This lateralization process can occur in a relatively short time period or remain stable [7].

Numerous studies suggest that connexin lateralization can be proarrhythmic. Irregular distribution of connexin 43 (CX43), together with low CX43 expression, has been related to arrhythmia generation due to abnormal propagation of the electrical impulse [8,9,10]. Both higher lateralization and lower expression of CX43 have been shown in diseased hearts as compared to healthy hearts [11,12]. On the other hand, other studies postulate that connexin lateralization may compensate for cardiac inhomogeneities [13]. Additionally, there is evidence of heterocellular connections between CMs and fibroblasts through connexins [14,15,16]. These connexions usually occur on the lateral sides of the myocytes and could have a protective role.

A common limitation of most studies assessing morphological properties of cardiac tissue is the relatively small area that is evaluated to assess the high number of microscopic connections. Since manual quantification is extremely hard and time-consuming, the amount of tissue that can be analyzed is usually a very limited portion of the heart. Relatively new detection methods have been proposed for cellular and molecular analysis from experimental images, including some based on artificial intelligence [17,18,19] and the use of convolutional neural networks [20]. In glioma cell cultures, an automated method for quantification of cell-cell coupling has been reported [21]. In rat ventricular tissues, a quantitative approach has been published for the analysis of gap junction distribution [22]. Other quantitative approaches have been described that use co-localization of CX43 with N-Cadherin in rat and human atria [23]. Still, successful assessment of human cardiac tissue requires further efforts. So far, no fully automatic methods for detection and morphological characterization of CMs’, as well as for quantification and localization of CX43, have been described.

In this work, we present an automated quantitative software to measure CM dimensions and to evaluate CX43 amount and distribution. We analyze human and rat myocardial tissue sections based on automatic mask generation of myocytes from images of fluorescence immunohistochemistry. The generated mask is subsequently contoured and the contour of each CM is enclosed in a rectangle of minimum area. The use of morphological filters allows ensuring plausible myocyte characteristics. The generated rectangles are splitted into four equal parts, two at the CMs’ longitudinal ends and two in the middle of them, from which lateral-to-total CX43 ratio is determined. The performance of our proposed algorithm is shown to be comparable to that of a human expert, with a highly relevant advantage of our algorithm in terms of computation time. To improve the quality of the output, supervised and manual modes are additionally implemented. A stand-alone version of our software Myocyte Automatic Retrieval and Tissue Analyzer (MARTA) is available for the research community (https://github.com/tonibois/MARTA). A set of supporting video demonstrations for MARTA usage are additionally available (Supplementary Materials, MARTA guide).

## 2. Materials and Methods

### 2.1. Sample Collection and Fluorescent Immunohistochemistry

Human and rat ventricular tissue images obtained by using various combinations of antibodies and fluorophores and imaged with different microscopes were analyzed to show the versatility of our proposed software.

Tissue specimens of the human left ventricular wall or papillary muscle were collected at Hospital Universitario Miguel Servet (Zaragoza, Spain). The study was approved by the ethical committee (CEICA, reference number 05/2017) and all patients gave informed consent. Left ventricular biopsies were obtained using a 14G tru-cut needle during cardiac surgery, immediately after the patient was placed on cardiopulmonary bypass. Papillary muscles were resected during valve replacement using a razor blade. Immediately upon collection, tissue specimens were fixed in 4% paraformaldehyde solution for 1 h at 4 ${}^{\circ }$C and embedded in paraffin blocks. Tissue samples presenting longitudinal orientations were used for subsequent analysis.

**Figure 1:**
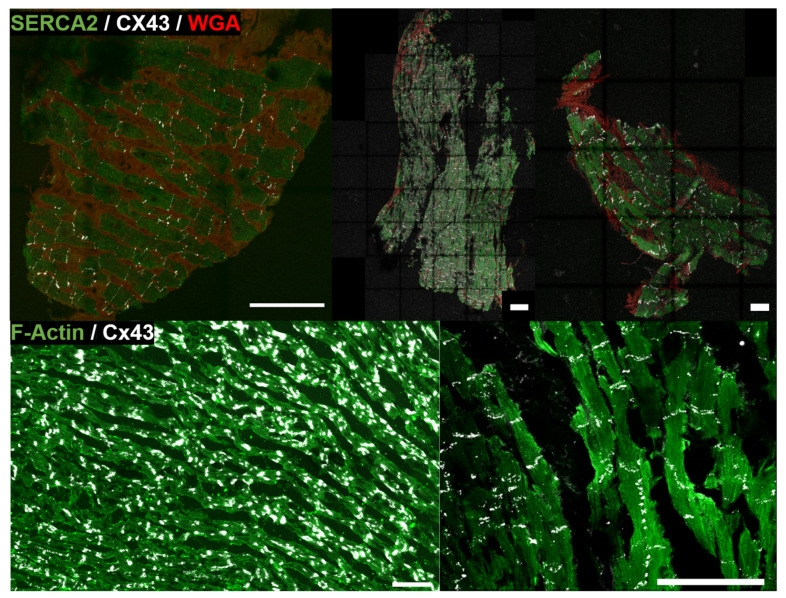
Input fluorescence microscopy images. *A*, *B*, *C*, from left to right: human samples from three individuals *a*, *b* and *c*. *D* and *E*, from left to right: rat samples d2 and *e*. Green signal corresponds to sarco/endoplasmic reticulum $Ca^{2+}$ ATPase (SERCA2) in humans and F-actin in rat samples (channel $c_{1}$). White signal marks connexin 43 (CX43) (channel $c_{2}$). Red signal is wheat germ agglutinin (WGA) in human samples (channel $c_{3}$). Scale bar is in all cases 100 μm.

Sections of 5 μm thickness were stained following standard immunohistochemical procedures. Primary antibodies were mouse anti-sarco/endoplasmic reticulum $Ca^{2+}$ ATPase (SERCA2) (ab2817, Abcam, Cambridge, UK) and rabbit anti-CX43 (ab11370, Abcam, Cambridge, UK), while secondary antibodies were Alexa Fluor 488 goat anti-mouse (A11029, ThermoFisher, Madrid, Spain) and Alexa Fluor 633 goat anti-rabbit (A21071, ThermoFisher, Madrid, Spain). The anti-human CX43 antibody was raised against a human peptide located after the N-terminal connexin conserved domain and, therefore, cross reactivity with other connexins is not expected. SERCA2 is located in the sarcoplasmic reticulum while CX43 is located in the sarcolemma. The extracellular matrix was stained with alexa-fluor 555 wheat germ agglutinin (WGA) (W32464, ThermoFisher, Madrid, Spain) to delimit the perimeter of the cells. Tissue sections were imaged using confocal microscopy Carl Zeiss LSM 880 (Carl Zeiss, Jena, Germany). The human tissue sections used in this study to evaluate the performance of our proposed methods as compared to a trained expert’s criteria are shown in panels A, B and C of Figure 1.

Hearts from 3-month-old, male Wistar rats were obtained and frozen in liquid nitrogen as described in [7]. For immunodetection of CX43 distribution from isolated rat hearts, we used 10-μm-thick left ventricular cryosections. Tissue sections were washed in phosphate buffer saline (PBS), fixed in ice-cold methanol, permeabilized in 0.3% Triton X-100 in PBS and blocked with the solution of 1% bovine serum albumin in PBS.

**Figure 2:**
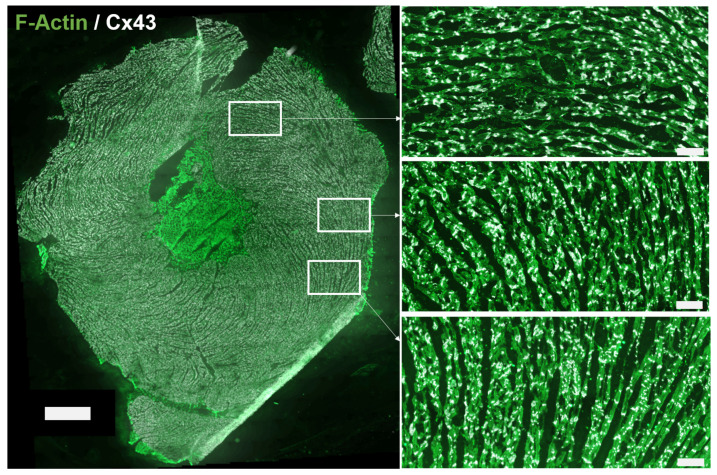
Image of a left ventricular rat tissue partly analyzed in this study (**left**) and three insets at higher magnification (**right**). Green signal represents F-actin (channel $c_{1}$). White signal is CX43 (channel $c_{2}$). Within the myocardium, black background estimates the interstitium (channel $c_{3}$). Scale bar: left, 1000 μm; right insets, 100 μm.

Primary mouse anti-CX43 antibody (MAB 3068, Chemicon International, Inc., Temecula, CA, USA) and fluorescence staining against F-actin (ab112124, Abcam, Cambridge, UK) were used. As per in humans, the anti-rat CX43 antibody was raised against a peptide located near the C-terminus, after the N-terminal connexin conserved domain, and no cross-reactivity with other connexins is expected. The secondary antibody was goat anti-mouse fluorescein isothiocyanate (FITC) (111-095-003, Jackson Immuno Research Labs, West Grove, PA, USA). After several PBS washes, tissues were mounted in the Vectashield medium (H-1200, Vector Laboratories-Inc., Burlingame, CA, USA) and captured by Axio Imager Z2 microscope equiped with ApoTome.2 (Carl Zeiss, Jena, Germany). The rat tissue sections used in this study for evaluation of our methods are shown in panels D and E of Figure 1, which are labeled as d2 (left) and *e* (right). Also, an illustration of a larger piece of rat tissue from which the image in the bottom left panel of Figure 1 was taken is presented in Figure 2. Details of the complex distribution of CX43 can be appreciated in the corresponding insets.

For both human and rat images, negative controls were considered by staining tissue sections with only the secondary antibodies in single or multiplex format. Fluorescence signal spillover between channels was tested negative in both species.

The input format for image analysis was TIFF. All TIFF images were exported from the Carl Zeiss Image (CZI) data format obtained by confocal fluorescence microscopy of cardiac tissues. In the case of human tissues (images *a*, *b* and *c* in panels A, B and C of Figure 1), TIFF images corresponding to each of the three fluorescence channels of SERCA2, CX43 and WGA were available, while in the case of rat tissues (images d2 and *e* in panels D and E of Figure 1), only the merge of CX43 and F-actin channels was available. Image d2 was also analyzed with only the F-actin channel (d1). Channel $c_{1}$ was used for CM regions (either marked by SERCA2 or F-actin), $c_{2}$ for CX43 and $c_{3}$ for interstitium. The merged images available from rat tissues were split to generate $c_{1}$, $c_{2}$ and $c_{3}$.

### 2.2. From Fluorescence Images to Cardiomyocyte Binary Masks

The first step of our proposed method was the conversion of the input images into grayscale by averaging of red, green and blue intensities. In some instances, an optional equalization of the histogram was subsequently applied onto each input channel (see Table 1 for specification of when histogram equalization was performed).

The next step was binarization of each *i*-th input channel $c_{i}$ using 8-bit binary thresholds $thr_{ci}$, thus reducing the grayscale color dimension to white and black (i.e., 1 and 0) masks $M_{ci}$:

(1) $$M_{ci}=\left\{\begin{array}{cc} 1 & if\;c_{i}>thr_{ci}\\ 0 & otherwise. \end{array}\right.$$

The values chosen for the parameters $thr_{ci}$ are provided in Table 1.

Upon binarization, morphological transformations were applied to $M_{ci}$, as described in the following. First, erosion followed by dilation (i.e., first removing and subsequently adding pixels on object boundaries), all together called opening operation, was applied to reduce the noise level by using a squared noise removal window of rank $n_{r,ci}$. While erosion removes the boundaries of isolated foreground objects, dilation compensates for erosion in large foreground objects. This results in deletion of small isolated objects usually associated with noise. Next, a dilation operation was applied to enhance boundaries by using growth windows of rank $n_{g,ci}$. The dilation operation was applied $n_{g,it,ci}$ times to the foreground boundaries of the binary mask objects. When $n_{r,ci}=n_{g,ci}=1$ was set, no transformation was applied to $M_{ci}$. A duplicate copy of $c_{2}$, denoted by $c_{4}$, was equalized and binarized using a fixed threshold value ($thr_{c4}=254\approx 99\%$) to obtain the mask $M_{c4}$, which was used for CX43 quantification and was treated without noise removal and without dilation ($n_{r,ci}=n_{g,ci}=1$). The values chosen for all the above described parameters are provided in Table 1.

After application of channel grayscaling, binarization, noise removal and growing, the binarized channels $M_{ci}$ were combined to render an automatic CM mask $M_{a}$ that was later compared against a manually generated mask $M_{m}$. The mask $M_{a}$ was generated by activating $M_{c1}$ if it did not intersect with $M_{c2}$ or $M_{c3}$ (i.e., pixels with a value of 1 in the operation $M_{c1}\;\bar{M_{c2}}$ or in the operation $M_{c1}\;\bar{M_{c3}}$, where $\bar{M_{c2}}$ and $\bar{M_{c3}}$ are the complementary images of $M_{c2}$ and $M_{c3}$, respectively).

The manual CM mask $M_{m}$ was generated by a biologist with expertise in the field using Krita 4.2.9 software (Krita’s GNU GPL license), who delineated CMs’ cell boundaries by visual inspection.

### 2.3. Cardiomyocyte Detection and Morphological Characterization

On the automatic and manual CM masks, $M_{a}$ and $M_{m}$, cell contouring was performed by a border following technique as described in [24].

Only the external contours were retrieved whereas child or internal contours were removed. After contouring, a filter was applied to select only plausible contour areas ($A_{cont}>$ 100 μm${}^{2}$) and perimeters ($P_{cont}>$ 40 μm). This step contributed to increase the processing speed by discarding contours unlikely to represent CMs. Next, each filtered contour was fitted into a rotated rectangle of minimum area.

By measuring the lengths of the rectangle’s long and short sides, a set of CMs’ morphological properties were calculated, including length (*L*), width (*W*), aspect ratio (R=L/W) and box area (A=L·W). The length *L* of the CM was computed from the longer side of the rectangle, but, rather than directly considering the fitted rectangle, further processing was applied (as described in the next section) and *L* was calculated by averaging the lengths of the original rectangle and an expanded version of it. The width *W* of the CM was computed as the value of the shorter side of the rectangle. A second filtering was applied to select only CMs with plausible lengths and widths according to: 20<L<200 μm, 5<W<50 μm. The filtered rectangles in $M_{a}$ and $M_{m}$ were denoted by $B_{a_{j}}$ and $B_{m_{i}}$, respectively. Their associated areas were correspondingly denoted by $A_{a_{j}}$ and $A_{m_{i}}$.

The width and length were converted from pixels to μm according to the scale-bar calibration, which was 0.210 μm/pixel for human ventricular images *a*, *b* and *c*, 0.227 μm/pixel for rat ventricular images derived from *d* (i.e., d1 and d2) and 0.114 μm/pixel for rat ventricular image *e*. Correspondingly, squared scale conversions were used to express areas in μm${}^{2}$/pixel${}^{2}$.

All the above described processing methods are part of the unsupervised, fully automatic mode of MARTA software. A supervised mode was additionally implemented, in which a pop-up window is displayed showing every detected CM and allowing the user to select the ones to be saved.

### 2.4. Quantification of CX43 Expression

The expression and lateralization of CX43, denoted by $C_{ex}$ and $C_{lat}$, were computed from the binarized CX43 channel $M_{c4}$. To quantify $C_{ex}$, the number of activated pixels in $M_{c4}$ was divided by the number of activated pixels in the tissue mask $M_{t}$. The mask $M_{t}$ was defined to take a value of 1 for pixels activated in the binarized channels $M_{c1}$, $M_{c2}$ or $M_{c3}$ and 0 otherwise.

If $N_{pix}$ denotes the total number of pixels in the image, $C_{ex}$ was calculated as the percentage given by:

(2) $$C_{ex}=100\cdot \frac{\sum_{j=1}^{N_{pix}}M_{t,j}\;M_{c4,j}}{\sum_{j=1}^{N_{pix}}\;M_{t,j}}\;,$$

where $M_{t,j}$ and $M_{c4,j}$ denote the value of pixel *j* in the mask $M_{t}$ and in the binarized channel $M_{c4}$, respectively.

A level of noise in the image was estimated by calculating the percentage of activated pixels in the binarized CX43 channel $M_{c4}$ that are not activated in the tissue mask $M_{t}$:

(3) $$r=100\;\frac{\sum_{j=1}^{N_{pix}}\bar{M_{t,j}}\;M_{c4,j}}{\sum_{j=1}^{N_{pix}}\;\bar{M_{t,j}}}\;.$$

### 2.5. Quantification of CX43 Distribution

**Figure 3:**
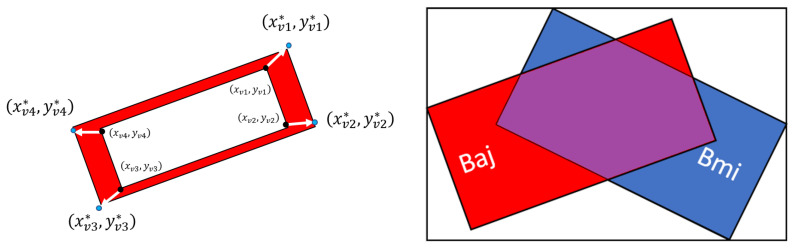
Left panel: representation of vertex projection from original coordinates ($x_{v,i},y_{v,i}$) to extended coordinates ($x_{v,i}^{*},y_{v,i}^{*}$). Right panel: illustration of how the intersection between cardiomyocytes (CMs) in the manual and automatic masks was computed. The red box $B_{a_{j}}$ shows the enclosing rectangle for a CM in the automatic mask $M_{a}$, whereas the blue box $B_{m_{i}}$ shows the enclosing rectangle for a CM in the manual mask $M_{m}$. The purple area is the intersection between the areas of the two rectangles: $A_{a_{j}}\;\;\cap A_{m_{i}}$.

Distribution of CX43 into polar (i.e., end-to-end) and non-polar (i.e., lateral) components incorporated an initial step where the rectangles fitted to each cell contour were expanded by using a box padding parameter *h*. Specifically, box padding was performed by projecting the vertex coordinates $\left(x_{vi},y_{vi}\right)$ of the fitted rectangle onto an outer direction according to a distance of *h* units. This is illustrated in the left panel of Figure 3. For the *i*-th vertex of the rectangle, with coordinates $\left(x_{vi},y_{vi}\right)$, we calculated the coordinates $\left(x_{vi}^{*},y_{vi}^{*}\right)$ of the expanded rectangle by using Thales theorem:

(4) $$\left(x_{vi}^{*},y_{vi}^{*}\right)=\left(x_{vi}+\frac{h(x_{vi}-x_{vi+2})}{d_{i,i+2}},\;y_{vi}+\frac{h(y_{i}-y_{vi+2})}{d_{i,i+2}}\right)$$

where $d_{i,i+2}$ is the distance between the *i*-th vertex, with coordinates $\left(x_{vi},y_{vi}\right)$, and the (i+2)-th vertex, with coordinates $\left(x_{vi+2},y_{vi+2}\right)$, where vertices 1 and 3 are related to each other as *i* and i+2, and analogously for vertices 2 and 4. The value of *h* was set to compensate for CM removal when $M_{c2}$ growth was applied with a window of rank $n_{g,c2}$ and a number of iterations $n_{g,it,c2}$: $h=2\cdot n_{g,c2}\cdot n_{g,it,c2}$. That compensation is proportional to the growth of the connexin mask dilation, since the larger the amount of growth applied to CX43, the more the quantity of cell mask is removed.

As mentioned in the previous section, the length *L* of each CM was obtained as the average of the long side of the fitted rectangle and the long side of the expanded rectangle.

After application of box padding, each CM was divided into four parts with respect to the longer side of the rectangle. The polar (end-to-end) compartments were denoted by $P_{0}$ and $P_{3}$, while the middle compartments were denoted by $P_{1}$ and $P_{2}$. The relative amount of CX43 in each compartment $P_{i}$, expressed as a percentage, was estimated by computing the number of activated pixels in the intersection between $P_{i}$ and $M_{c4}$ by the total amount of pixels $N_{i}$ in $P_{i}$:

(5) $$F_{i}=\frac{\sum_{j\in H_{i}}P_{i,j}\;M_{c4,j}}{N_{i}}\;.$$

where $H_{i}$ denotes the set of pixel indices in compartment $P_{i}$, *i* = 1, 2, 3, 4.

From these magnitudes, the relative amounts of polar and lateral CX43 were determined as $F_{p}=F_{0}+F_{3}$ and $F_{l}=F_{1}+F_{2}$, respectively. The percentage of CX43 lateralization, denoted by $C_{lat}$, was computed as:

(6) $$C_{lat}=100\;\frac{F_{l}}{F_{l}+F_{p}}\;.$$

A value of 0 for $C_{lat}$ indicated no lateral CX43, whereas a value of 100 indicated that all CX43 was located at the intermediate compartments, with no polar contribution.

A theoretical expression characterizing the frequency distribution of $C_{lat}$ for the pooled measures from the three images of human ventricular tissues (*a*, *b* and *c*) was calculated by fitting an exponential function to the $C_{lat}$ histogram:

(7) $$f\left(x\right)=0.03425\;e^{-0.033x}\;.$$

Integration of the function f(x) provides quantification for the amount of CMs with $C_{lat}$ values in a given range from $C_{lat_{1}}$ to $C_{lat_{2}}$:

(8) $$P\left(C_{lat_{1}}<x<C_{lat_{2}}\right)=\int_{C_{lat_{1}}}^{C_{lat_{2}}}\;f\left(x\right)\;dx\;.$$

### 2.6. Performance Evaluation

The performance of our proposed algorithm was contrasted with that of manual evaluation in terms of CM delineation, morphological characterization and CX43 distribution.

Specifically, the agreement was evaluated by comparison of measurements of *L*, *W*, *R*, *A*, $C_{lat}$ and Area Under the Curve (AUC), with AUC computed from the integrated percentile distribution of maximum intersection between manual and automatic masks as described in the following.

For each CM *i* of the manual mask $M_{m}$, the maximum intersection $I_{i}$ was computed by identifying the CM in the automatic mask $M_{a}$ having the largest area contained in it. For this computation, the intersection between the enclosed rectangle of the CM *i* in $M_{m}$ and any CM *j* in $M_{a}$ was first computed as:

(9) $$I_{i,j}=100\frac{A_{m_{i}}\;\cap \;A_{a_{j}}\;}{A_{m_{i}}}\;.$$

$I_{i,j}$ ranges from 0 when there is no intersection between CMs i and j to 100% when there is full overlapping. Subsequently, $I_{i}$ was computed as the maximum value of all $I_{i,j}$ values for *j* covering all CMs in mask $M_{a}$.

If $N_{a}$ denotes the number of CMs in mask $M_{a}$, $I_{i}$ was computed as:

(10) $$I_{i}=max{\left(I_{i,j}\right)}_{j=1,..,N_{a}}\;.$$

Once $I_{i}$ was obtained for all CMs in $M_{m}$, the corresponding percentile curve $P_{c}\left(k\right)$ was computed for each *k*-th integer percentile from 1 to 100. To compute AUC, $P_{c}\left(k\right)$ was integrated numerically using the trapezoidal rule:

(11) $$AUC=\frac{1}{10^{4}}\sum_{k=1}^{100}\frac{P_{c}\left(k\right)+P_{c}\left(k+1\right)}{2}$$

## 3. Results

### 3.1. Automated Image Analysis

**Figure 4:**
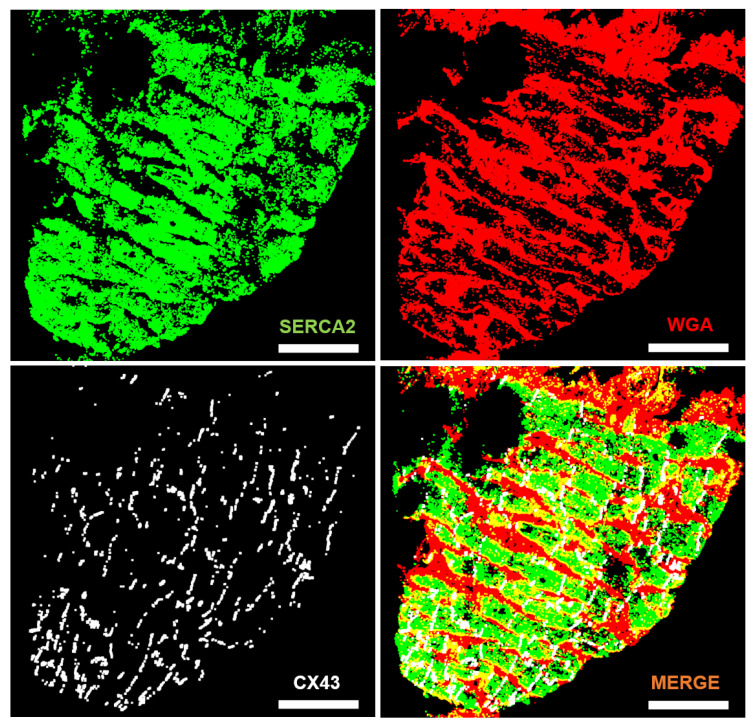
Top: $M_{c1}$ (SERCA2) and $M_{c3}$ (WGA) binarized channels. Bottom left: $M_{c2}$ (CX43) binarized channel. Bottom right: merged channel. Scale bar = 100 μm.

The values of the parameters used to generate the automatic masks $M_{a}$ from input images *a*, *b*, *c*, d1, d2 and *e* are summarized in Table 1. Input images d1 and d2 correspond to the same image *d* but in the first case using one channel (F-actin) and in the second case using two channels (F-actin and CX43). As it can be observed from the values of the configuration parameters used for images *a*, *b* and *c*, techniques for noise removal and growing were applied to channels $M_{ci}$, i∈{1,2,3}. This was performed by setting values above 1 to the parameters $n_{r,ci}$, $n_{g,ci}$ and $n_{g,it,ci}$, i∈{1,2,3}. The binarized channels obtained for image *a* are presented in Figure 4.

**Figure 5:**
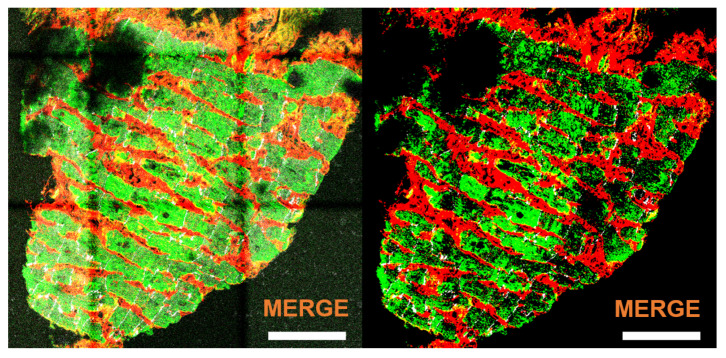
Merged channels for image *a* before (**left**) and after (**right**) noise removal. Scale bar = 100 μm.

The results of applying techniques to deal with noise in the input images are illustrated in Figure 5. Since the input images may present brightness variations, this may cause loss of information in dark tissue areas having values below the established value for the binarization threshold ($thr_{ci}$). If the threshold value were increased to capture a larger amount of tissue, a high level of noise might be present in the binarized channels (see Figure 5, left panel). In this study, noise removal was performed (Figure 5, right panel). As this operation may remove part of the tissue too, dilation was subsequently applied to compensate for those effects. Figure 4, bottom right panel, shows the result of all the processing steps applied to image *a*.

### 3.2. Agreement between Automatic and Manual Cell Delineation

**Figure 6:**
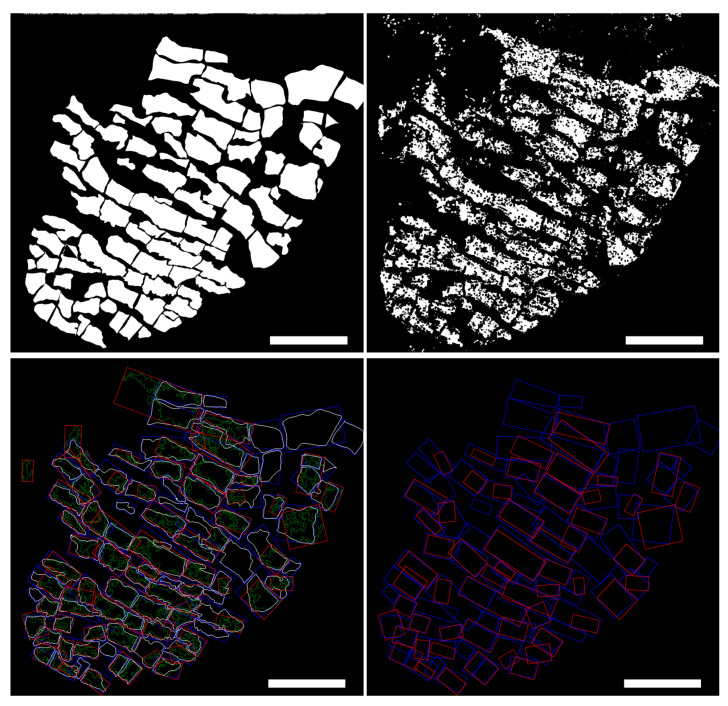
Manual versus automatic cell delineation in image *a*. Top left: mask $M_{m}$ obtained by manual delineation of CMs’ boundaries in image *a*. Top right: mask $M_{a}$ generated by Myocyte Automatic Retrieval and Tissue Analyzer (MARTA) software for image *a*. Bottom left: CMs’ contours for manual (white) and automatic (green) masks and corresponding enclosing rectangles (blue for manual, red for automatic). Bottom right: Enclosing rectangles from manual and automatic masks showing overlapping above 50%. Scale bar = 100 μm.

Figure 6 illustrates the results of the comparison between the manually delineated mask $M_{m}$ (top left panel) and the computed automatic mask $M_{a}$ (top right panel) for image *a*. The cell contours and the fitted rectangles for both $M_{m}$ and $M_{a}$ are shown in the same figure (bottom left panel).

The agreement between masks $M_{m}$ and $M_{a}$ can be appreciated from the bottom right panel of Figure 6, which shows all CMs *i* in $M_{m}$ that match one CM in $M_{a}$ with overlapping $I_{i}>$ 50%. For image *a*, 66 CMs were detected in $M_{a}$ as compared to 84 CMs detected in $M_{m}$. For image *b*, 453 CMs were detected in $M_{a}$ and 371 in $M_{m}$. For image *c*, corresponding numbers were 82 for $M_{m}$ and 64 for $M_{a}$. Full details on the number of detected CMs in other input images, namely d1, d2 and *e* from rat ventricular tissue, are provided in Table 1.

**Figure 7:**
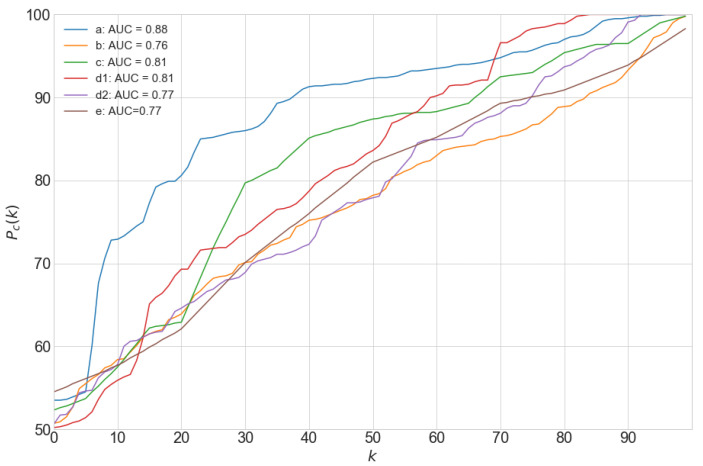
Percentile curves of maximum intersection $I_{i}$ for CMs *i* in $M_{m}$, for all the analyzed images. Area Under the Curve (AUC) values are presented in the legend.

In Figure 7, the performance of the automatic algorithm is shown in terms of the percentile curve $P_{c}\left(k\right)$ for CMs sharing more than 50% overlap in manual and automatic masks. For the standard set of parameter values used in this study, the best algorithm performance was obtained for image *a* with an AUC of 0.88. For images *b* and *c*, the algorithm performances were similar, with AUC values of 0.76 and 0.81, respectively. The number of CMs overlapping more than 50% in the manual and automatic masks was 58 for image *a* (69% of detected CMs in $M_{m}$), 172 (46%) for image *b* and 21 (26%) for image *c*.

In the rat ventricular samples, the performance was better for image d1 (AUC = 0.81) than for image d2 (AUC = 0.77), with low proportions of CMs highly overlapping in manual and automatic masks (19% and 29%, respectively). For image *e*, AUC was 0.77 and the corresponding percentage of CMs was 25%.

### 3.3. Cardiomyocytes’ Morphological Measurements

**Figure 8:**
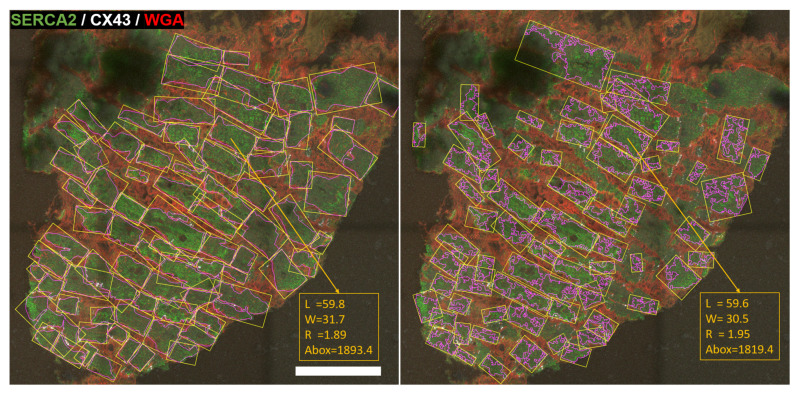
CMs in the manual (**left**) and automatic (**right**) masks contoured in pink, with enclosing rectangles in yellow. At the bottom right corner of each panel is an example of morphological measures computed for a CM detected in both masks. Scale bar = 100 μm.

An example of all CM detections in automatic and manual masks $M_{a}$ and $M_{m}$ for image *a*, as well as individual measurements of morphological properties for a given CM, are presented in Figure 8. As it can be observed from the figure, there is overall agreement between the CMs identified automatically and manually, although there are some CMs, particularly at the borders of the tissue, where the two methods disagree. For the CM for which morphological measurements are shown in the figure, similar lengths and widths are rendered by both methods.

**Figure 9:**
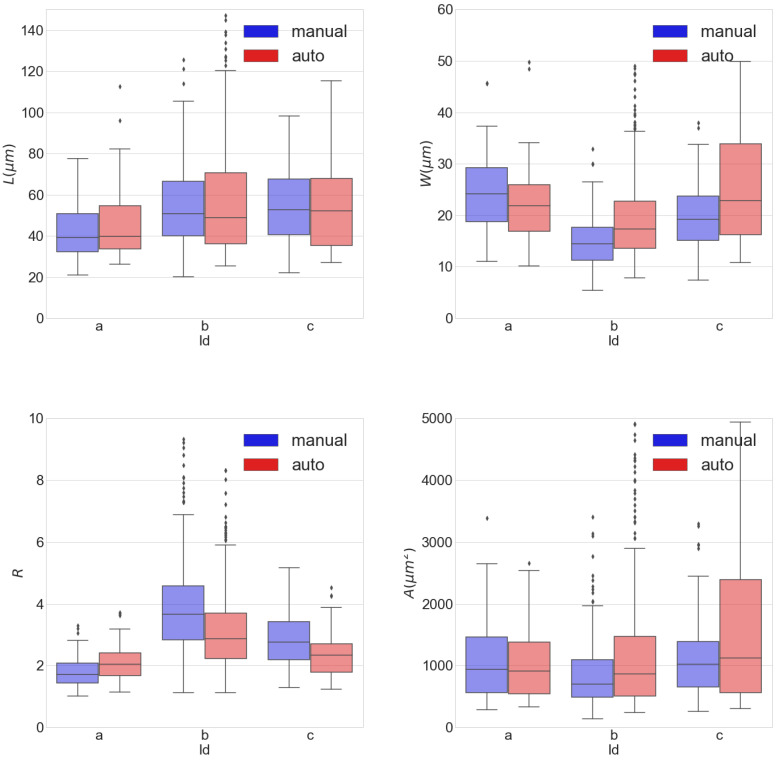
Box plots of length *L* (top left), width *W* (top right), length-to-width ratio *R* (bottom left) and area *A* (bottom right) for manual (in blue) and automatic (in red) masks, calculated for images *a*, *b* and *c*.

In Figure 9, box plots showing statistical results for *L*, *W*, *R* and *A* are shown for images *a*, *b* and *c*. As it can be appreciated from the figure, the statistical measures for all morphological markers obtained by the automatic algorithm are in line with those measured manually. The interquartile ranges for *L* in $M_{a}$ and $M_{m}$ span from 20 to 120 μm, while those for *W* span from 5 to 50 μm. This translates into interquartile ranges for *R* covering from 1 to 7.

### 3.4. Quantification of CX43 Expression

**Figure 10:**
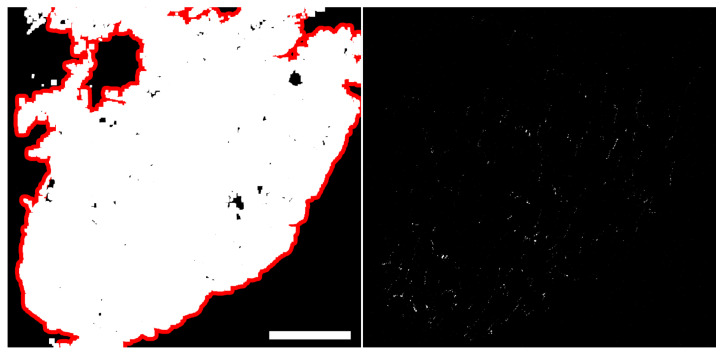
Left: Tissue mask $M_{t}$ represented in white, enclosed by maximum contour in red, for image *a*. Right: Equalized and binarized channel $M_{c4}$ ($thr_{c4}=254$) for the same image. Scale bar = 100 μm.

Figure 10 illustrates the tissue mask $M_{t}$ obtained by combining channels $M_{c1}$, $M_{c2}$ and $M_{c3}$ (left panel) and shows the binarized channel $M_{c4}$ (right panel) for image *a*. $C_{ex}$ for this example was 0.27%, which was obtained by dividing the number of activated pixels in $M_{c4}$ by the number of activated pixels in $M_{t}$.

Table 2 presents the results for $C_{ex}$ obtained by using binarization with different selections of threshold values. The total tissue areas for images *a*, *b* and *c* were 0.12, 0.76 and 0.17 mm${}^{2}$, respectively.

If histogram equalization of $M_{c4}$ is performed, values of CX43 below 1.6% are obtained when binarizing $M_{c4}$ using $thr_{c4}$ = 253 (i.e., 98% to 100%, see Equation (1)). For $thr_{c4}$ = 253, a high level of noise is still included (Table 2). Thus, $thr_{c4}$=254 was selected for quantification of CX43 expression and lateralization, which provides a good compromise between noise levels (r< 0.1%) and CX43 expression ($C_{\mathrm{ex}}<1.6\%$).

### 3.5. Determination of CX43 Distribution

**Figure 11:**
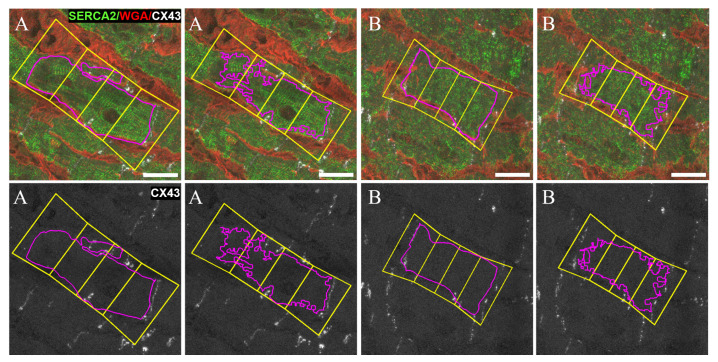
Representative images of box partitioning for two CMs A and B of the manual $M_{m}$ (**left**) and automatic $M_{a}$ (**right**) masks. Top: overlay of channels $c_{1}$ (SERCA2, green), $c_{2}$ (CX43, white) and $c_{3}$ (WGA, red) and results of CMs contour in pink, and the minimum area rectangles enclosing them in yellow. Rectangles are divided into four regions corresponding to polar (i.e., end-to-end) and lateral (i.e., middle) cell compartments. Bottom: channel $c_{2}$ without binarization. Polar CX43 bands appear as discontinuous line patterns because of the low signal intensity. Scale bar = 20 μm.

Examples of two different CMs (A and B) that were box partitioned for $C_{lat}$ quantification by both manual and automatic methods (Figure 11). CM A presents an extremely high lateral-to-total CX43 ratio ($C_{lat}$ = 79.4%, in $M_{m}$) while the result in $M_{a}$ is similar for the same CM ($C_{lat}$ = 73.9%). On the other hand, CM B has a lateral-to-total CX43 ratio close to the global average, being $C_{lat}=$ 24.4% for $M_{m}$ and $C_{lat}$ = 23.0% for $M_{a}$.

**Figure 12:**
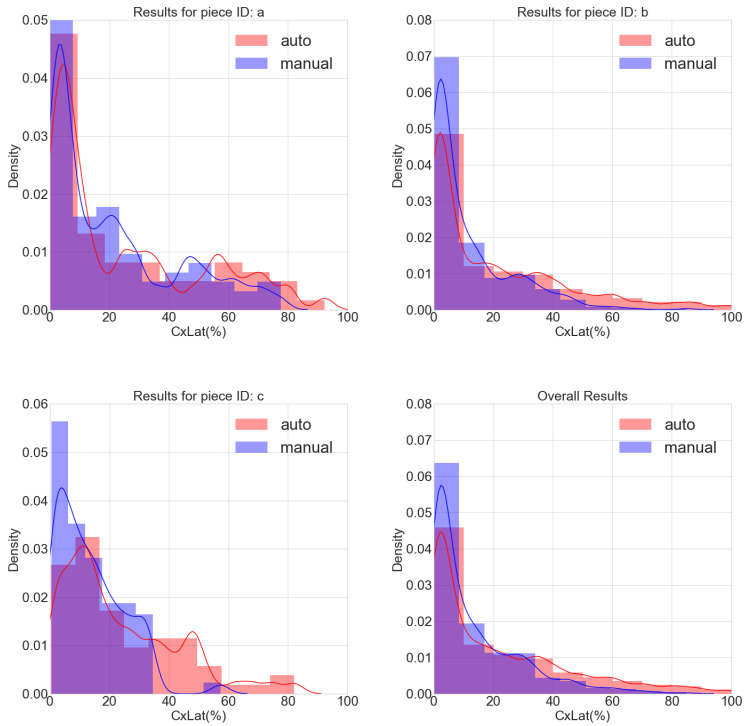
Histograms and density plots for $C_{lat}$ computed for images *a* (**top left**), *b* (**top right**) and *c* (**bottom left**), as well as for pooled data from the three images (**bottom right**), both from automatic (red) and manual (blue) CM masks.

The results from the analysis of the full images *a*, *b* and *c*, as well as pooled results from the three images together, are presented in Figure 12. As it can be observed from the bottom right panel showing overall results, a large number of CMs present low values of $C_{lat}$, with the curve fitted to the frequency distribution decaying exponentially as $C_{lat}$ increases (Equation (7)). From this fitted curve, we found that 50% of CMs had $C_{lat}<$ 20%. Also, 84% of CMs had higher polar than lateral CX43 expression (i.e., $C_{lat}<$ 50%).

### 3.6. Application to Images with One or Two Channels

**Figure 13:**
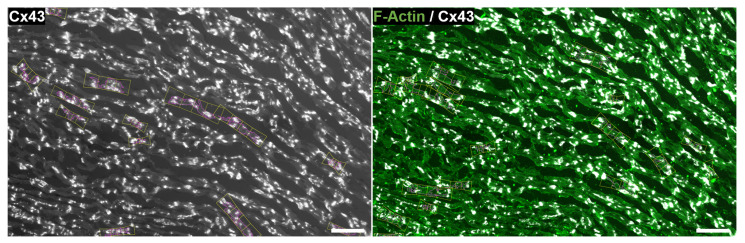
Detected CMs in the automatic mask $M_{a}$ computed under the supervised mode for images d1 (**left**) and d2 (**right**). Scale bar = 100 μm.

Figure 13 shows the result of processing images d1 (left) and d2 (right), corresponding to the same image *d* but with one and two channels, respectively. As can be seen from the figure, the contrast in d1 is low and the brightness is heterogeneous, which only allows CM detection in certain regions of the image. The number of detected CMs in d1 is 111 (28 in the supervised mode), while it is slightly increased in d2 to 170 (44 in the supervised mode).

**Figure 14:**
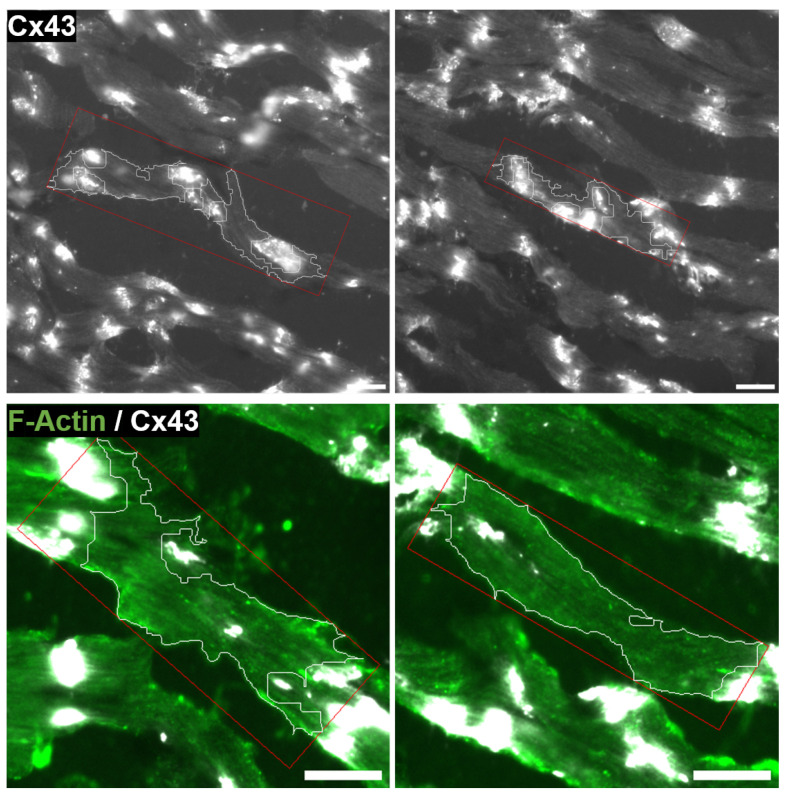
Examples of four detected CMs from automatic masks obtained for images d1 (**top**) and d2 (**bottom**). Morphological measurements and $C_{lat}$ values are provided in Table 3 for $d1_{CM,1}$ (**top left**), $d1_{CM,2}$ (**top right**), $d2_{CM,3}$ (**bottom left**) and $d2_{CM,4}$ (**bottom right**). Scale bar = 20 μm.

Examples of detected CMs in images d1 and d2 are displayed in Figure 14. Corresponding quantitative values for CMs’ morphological measurements and $C_{lat}$ are provided in Table 3. As can be seen from the table, the four illustrated CMs of images d1 and d2 have lengths above 100 μm. However, other CMs in the same image presented remarkably lower lengths. In mean over the whole image, *L* was 71 μm when measured from d1 and 73 μm when measured from d2, using the supervised mode. In the manual evaluation, the mean value of *L* was 73 μm. Average widths (W) were in the range from 23 to 25 μm for both manual and automatic methods. Also, high heterogeneity in $C_{lat}$ over CMs can be appreciated from the table. Mean $C_{lat}$ over the full image was 19.6% from manual evaluation, whereas results from automatic evaluation were 18.7% in supervised d1 and 13.5% in supervised d2.

**Figure 15:**
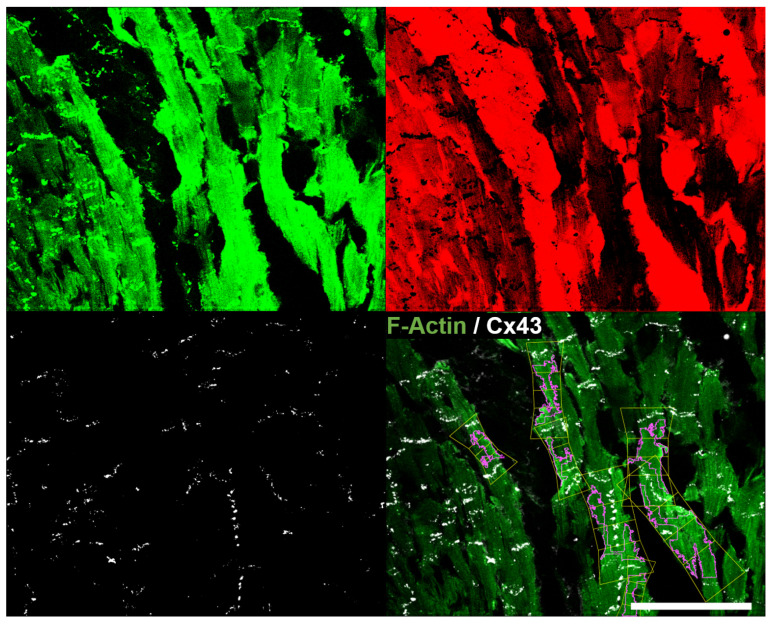
Binarized channels representing the CM mask $M_{c1}$ (**top left**, in green), the interstitium generated by inverting the CM mask (**top right**, in red) and the CX43 mask $M_{c2}$ (**bottom left**, in white), as well as original image *e* with overlaid CMs detected in supervised mode (**bottom right**). Scale bar = 100 μm.

Figure 15 presents the results of automatically processing image *e* in supervised mode. Considering the full image, 12 CMs were retrieved by the automatic algorithm (7 in supervised mode). The average length of the 7 retrieved CMs in image *e* is 60 μm, with average width of 24 μm and average $C_{lat}$ of 21%.

**Figure 16:**
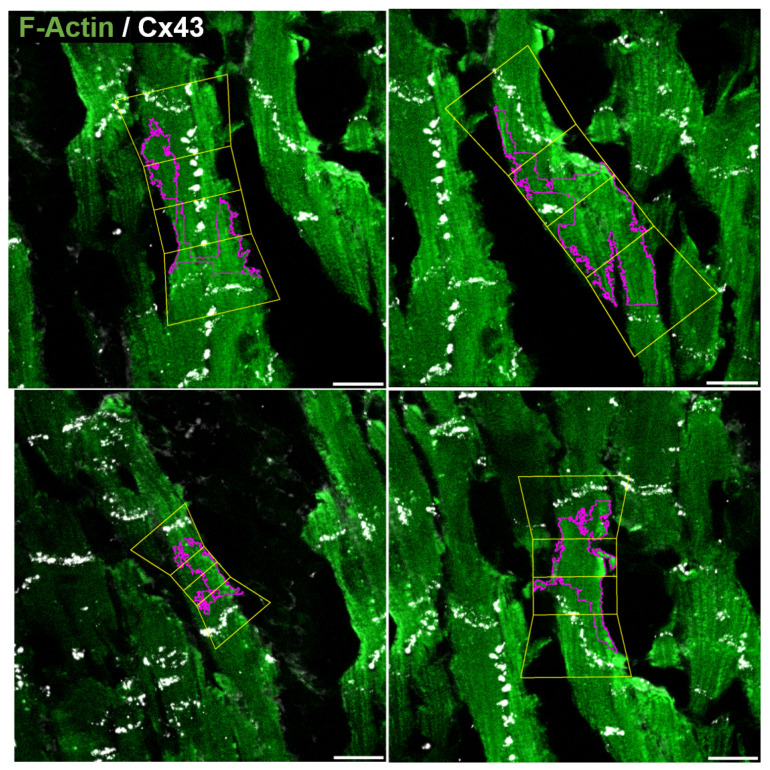
Four detected CMs from the automatic mask $M_{a}$ on top of image *e*. Morphological measurements and $C_{lat}$ values are provided in Table 3 for $e_{CM,1}$ (**top left**), $e_{CM,2}$ (**top right**), $e_{CM,3}$ (**bottom left**) and $e_{CM,4}$ (**bottom right**). Scale bar = 20 μm.

Particular examples of detected CMs are shown in Figure 16, with corresponding morphological measures provided in Table 3. The four illustrated CMs ($e_{CM,1}$ to $e_{CM,4}$) present lengths in the range of 50 to 120 μm, with *R* ratios varying from 2 to 4. It can be noted that two of the CMs ($e_{CM,1}$ and $e_{CM,2}$) have a high degree of lateralization with $C_{lat}$ values above 30%. For the first CM ($e_{CM,1}$), the large amount of CX43 in the lateral compartments is clear from the figure. Also, it should be noted that, despite the fact that two cells are contained in $e_{CM,1}$, the algorithm is still able to deal with correct $C_{lat}$ quantification, as both cells are aligned and the compartmentalization into polar and end-to-end contributions is well approached. For the second CM ($e_{CM,2}$), $C_{lat}$ may have been overestimated due to the automatic method accounting for polar regions in lateral parts in association with a larger curvature of the CX43 signal (see Figure 16). The third CM ($e_{CM,3}$) presents an averaged $C_{lat}$ value of 22% and the fourth one ($e_{CM,4}$) has practically all CX43 in the polar compartments (see Table 3).

### 3.7. Processing Time

The processing times required by our software for CM delineation as compared to those required for manual processing by a trained expert are presented in Table 4. Using a single i7-CPU, the automatic mask was generated for image *a* in 1 s, for image *b* in 15.4 s and for image *c* in 3.2 s. On average, the processing speed was 66 CMs per s for image *a*, 29 CMs per s for image *b* and 19 CMs per s for image *c*. This contrasts with the manual processing speed, which, on average, was of 0.11 CMs per s. On the other hand, the manual processing of 344 CMs in image *d* required around 50 min. This implies an average time of 8.7 s per CM. Consequently, the automatic method rendered between fifty and six hundred times faster delineation than the manual method.

The full processing of the analyzed images by our automatic algorithm, including CM delineation and morphological characterization, as well as quantification of CX43 expression and distribution, took 22 s for image *a*, corresponding to a processing speed of 3 CMs per s. For images *b* and *c*, the whole processing times were 1036 and 54 s and the processing speeds were 0.5 and 1 CMs per s. The full manual processing of $C_{lat}$ was performed in 17 CMs of image *e* in 2 h and 39 min. This is equivalent to a full manual processing speed of 0.0018 CMs per s.

## 4. Discussion

We have presented a methodology that allows automatic detection of CMs, as well as quantification of CX43 expression and lateralization from fluorescence microscopy images, as precisely as a manual method. Our software, MARTA, can run, on average, more than two hundred times faster than a human expert, being able to delineate CMs at a processing speed of at least 20 CM/s and performing the full processing of CMs’ morphological characterization and CX43 evaluation at a rate of 5 CM/s. Since both cell size and CX43 distribution are important factors in the propagation of the electrical signal in cardiac tissue, but their evaluation is time-consuming and requires training, MARTA is expected to find application in a wide variety of investigations.

### 4.1. Cardiomyocytes’ Morphological Measurements

Our MARTA software detected around 80% of manually delineated CMs from three-channel images of human ventricular tissues. This percentage was reduced when one- or two-channel images of rat ventricular samples were evaluated, globally leading to detection rates around 50%. Also, the accuracy in CM delineation was good.

AUC values ranged from 0.76 to 0.88 for both human and rat images, as evaluated from CMs overlapping at least 50% in manual and automatic masks.

In terms of CMs’ morphological measures, the average width of detected human ventricular CMs was around 20 μm when evaluated from the automatically generated mask and 18 μm from the manually delineated mask. These values are in good correspondence with those reported in the literature for human ventricular tissues [25]. The average length of human CMs was slightly above 50 μm, both when measured automatically and manually. This value is shorter than the ones reported in some studies, particularly those analyzing isolated CMs, where the average length was around 100 μm [25,26]. However, they are in good agreement with values reported in other studies [27,28]. It should be noted that the small size of our analyzed human tissue samples may have led to a relatively high proportion of CMs at the borders of the tissue. These CMs are more commonly hypercontracted as compared to the ones in the center of the tissue, thus reducing our reported averaged CM length. In any case, independently of the high variability in length measurements in the literature and the potential degree of tissue damage in the borders of the analyzed human samples, our software, MARTA, rendered length values that were in concordance with those obtained from manual delineation of the same tissue samples. Moreover, this was true for CMs’ width, length-to-width ratio and area as well, which confirms the capacity of our algorithm for automatic morphological characterization of CMs from fluorescence images.

For rat ventricular tissues, the average length of CMs found in this work was 74 μm, while the average width was 25 μm, for the algorithm working in the supervised mode. These values are in agreement with those obtained from manual evaluation, where the average length and width were 70 and 23 μm, respectively. Lower values of rat CMs’ width, but similar or slightly larger values of rat CMs’ lengths, have been reported in the literature [29]. As opposed to human images, the rat images analyzed in our study did not include WGA as a marker of connective tissue, which may have caused, in certain specific cases, the algorithm to consider two adjacent CMs as only one, thus contributing to a larger averaged width. This highlights the convenience of using interstitium-specific staining methods for precise morphometric analysis.

Despite the importance of objectively quantifying morphological characteristics of CMs, methods for automatic cell segmentation and shape characterization are scarce. In [30], an algorithm was developed for this purpose, which used images of nuclei and of the myocyte-specific cytoskeletal protein α-actinin. The algorithm included several image processing steps applied onto images from neonatal rat ventricular cultures, including some to remove non-myocyte cells and to identify cell boundaries based on a nuclear propagation approach. As opposed to the method in [30], our software did not use information from nuclei staining and could work with only the fluorescence channel of F-actin for rat ventricular tissues. However, as mentioned above, we confirmed that CM perimetral delimitation improved the software performance. The potential benefits of combining our developed methods with those proposed in [30], provided nuclei staining is available, should be assessed in further studies.

Based on the capacity of our MARTA software for the assessment of CMs’ morphological characteristics, we envision its potential application to different investigations, such as, e.g., the study of myocyte hypertrophy or atrophy from longitudinal or transversal sections of cardiac or even skeletal muscle tissue. In such a way, MARTA would allow characterization of cardiac hypertrophy present in different heart disorders and would also be applicable to studies investigating exercise-derived hypertrophy or disease-induced atrophy of skeletal muscle.

### 4.2. CX43 Expression and Distribution

To quantify CX43 expression, the binary mask $M_{c4}$ was used with a threshold set to $thr_{c4}=254$. The selection of this threshold was based on experimental results and theoretical estimation as described in the following. The expected percentage of CX43 with respect to CM area was estimated by measuring the width of CX43 bands. These widths were, on average, four pixels in a 0.21 μm/pixel scale resolution, thus representing 0.84 μm. This corresponds to ≈1.6% of the cell area for polar connexin contribution by assuming one continuous band per CM.

If accounting for lateral contributions of 20%, as previously reported [7], the global proportion of CX43 should be close to 2% of the cell area. Some studies have suggested that CM represents 80% of volume fraction [31], but that proportion would be lower for 2D longitudinal sections as compared to 3D volumes. Thus, 2% of CX43 per CM area would be translated into CX43-to-tissue fraction of 1.6% or less.

Thus, for instance, for CM lengths of 100 μm, which are roughly double the value found in this study, the expected $C_{ex}$ should be 0.8% or less. This condition, together with low noise level (r< 0.1%), could only be found by setting the binary threshold in the equalized $c_{4}$ channel to $thr_{c4}=254$.

By using the mentioned threshold, CX43 in polar and middle cell compartments was quantified by our method, leading to average values of lateral-to-total CX43 ratio of 19.7% for human and 19% for rat ventricular tissues. For human samples, the percentages of lateral CX43 from our automatic method are in concordance with the percentages obtained from the manually delineated masks. For rat samples, our results are consistent with those from manual evaluation and with percentages reported in previous studies [7]. Also, the average CX43 lateralization percentages obtained here are in line with those used in computational studies investigating the effects of cell-to-cell communication on cardiac electrical propagation [13]. Other studies have reported methods for analysis of CX43 distribution from cardiac tissue images. However, in some cases the method is not fully automatic but relies on manual segmentation of CMs [22]. Other methods require concomitant N-cadherin staining and rest on assumptions like CX43 being lateral only when it does not co-localize with N-cadherin [23]. Our method can work in a fully automatic (unsupervised) mode, as well as in a semi-automatic (supervised) mode, and can quantify CX43 distribution from merged images with one or two markers.

Provided that our automatic software, MARTA, can not only characterize CMs’ morphological measurements but also CX43 expression and distribution, we suggest it could be used as a tool to help in the development of detailed in silico models of cardiac electrophysiology. Our algorithm could take experimental fluorescence images as an input and provide CX43 profiling on delineated CMs as an output. These results could be incorporated into subcellular, cellular and tissue models to simulate cardiac electrical activity with higher precision in the definition of the transversal-to-longitudinal conductivity ratio and in spatial assignment of cell-to-cell communication based on the determined probability density distribution of CX43 from experimental images.

### 4.3. Additional Features of the Proposed Software

The MARTA software provides an optional graphical output to allow the user to decide whether results are deemed as acceptable, or whether an alternative processing using different parameter settings would be of interest. Also, it provides two working modes, one fully automatic (unsupervised mode) and another one (supervised mode) in which the user can select and store only those detected CMs that are considered as appropriate. Particularly for the unsupervised mode, no prior training is required, while for the supervised mode a minimal training phase would be desirable. Also, MARTA has the capability to generate manually delineated masks over merged input images and to process those generated masks as complementary features of the full automatic process. This capability allows improving the precision of the results obtained from the automated or supervised algorithm when these are deemed as not completely satisfactory.

One of the main advantages of the MARTA software is that it allows to process merged images. Despite not being the main focus of the present study, it is worth saying that it can perform relative estimations of myocardium and interstitial proportions in a tissue in a similar way as it does for CX43 quantification. MARTA also implements angular orientation of CM boxes and total tissue area estimations by counting the number of activated pixels in the generated tissue mask $M_{t}$. This total tissue area can be computed by estimation of the maximum contoured area in $M_{t}$ too. The second approach could be more precise if there is a significant number of non-activated pixels in tissue regions but also more expensive in terms of computational cost.

The developed software is not specific of ventricular tissue but could be applicable to evaluate CMs’ morphological characteristics, as well as CX43 expression and distribution in atrial tissue, too. Also, MARTA could find application in the study of lateralization of other proteins involved in cardiac conduction, like CX40 [32].

### 4.4. Study Limitations and Future Extensions

The results presented in this study were obtained for specific parameter settings. These settings, provided for both equalized and non-equalized channels, have been established as the default option in our software, MARTA. However, since they may not be the ones providing optimal outputs for other types of images, the user can decide whether other values should be set for some of the algorithmic parameters. If channel equalization is chosen, which is provided as an option in the software, the calibration task becomes simpler than when no equalization is applied.

In some challenging cases, as in our analysis of the rat tissue image d1 with only one fluorescence channel, the performance of our software was compromised. In that particular case, when we applied our software in the supervised mode, only 20% of myocytes were selected for further processing. On top of performing further research to improve the processing steps to deal with those cases, we strongly recommend first assessing the quality and brightness homogeneity of the input images, particularly to avoid out of focus regions and high brightness heterogeneity. In future studies, we plan to include additional pre-processing steps. These may include the use of multiple foci of the same image by Extended Deep of Field [33], with image equalization methods [34], histogram normalization [35] or application of global [36] or local dependent thresholding [37,38,39].

Also, it is advisable to include channels delimiting the cell perimeter (e.g., WGA to mark the extracellular matrix), as this helps in CM delineation and notably improves the obtained outcomes.

The MARTA software allows to automatically process relatively large amounts of data in a reduced amount of time and provide statistical analysis with potential use for cardiac tissue characterization. In this regard, our software could be used for systematic analysis of digital repositories. Due to the exponential growth of digital information and expectations of its continuous increase in the near future [40], automated solutions represent a main technological focus. As an extension of the present work, the development of algorithms based on deep neural networks could be a good choice to further improve the obtained results. These types of algorithms have gained increasing interest due to their high performance, usually overcoming human performance for very specific tasks.

## Figures and Tables

**Table 1:** Image characteristics (♯ input images, ♯ fluorescence channels) and values of parameters used for CM mask generation and CX43 quantification. Thresholds are set in 8-bit range ([0, 255]). Noise removal and growth parameters $n_{r,ci}$ and $n_{g,ci}$ are expressed as matrix rank, while $n_{g,ci,it}$ is expressed as number of iterations. The last two rows contain the number of detected CMs in the automatic ($N_{a}$) and manual ($N_{m}$) masks. For images *d* and *e*, the supervised mode was additionally used to filter the results of the automatic algorithm. The number of CMs detected in the supervised mode are presented in brackets next to the value of $N_{a}$ obtained in the unsupervised mode.

ID	*a*	*b*	*c*	d1	d2	*e*
♯ inputs	3	3	3	1	1	1
♯ channels	3	3	3	1	2	2
equalized (y/n)	n	n	n	y	y	y
supervised (y/n)	n	n	n	y	y	y
scale	0.21	0.21	0.21	0.227	0.227	0.114
$thr_{c1}$	8	8	8	128	100	70
$thr_{c2}$	15	15	15	254	254	254
$thr_{c3}$	2	2	2	128	100	70
$thr_{c4}$	254	254	254	254	254	254
$n_{r,c1}$	3	3	3	1	1	1
$n_{g,c1}$	3	3	3	3	3	2
$n_{g,it,c1}$	3	3	3	3	3	1
$n_{r,c2}$	3	3	3	1	1	1
$n_{g,c2}$	3	3	3	4	4	5
$n_{g,it,c2}$	5	5	5	8	8	20
$n_{r,c3}$	3	3	3	1	1	1
$n_{g,c3}$	3	3	3	3	3	2
$n_{g,it,c3}$	3	3	3	3	3	1
$N_{a}$	66	453	64	111 (28)	170 (44)	15 (7)
$N_{m}$	84	371	82	344	344	46

**Table 2:** Expression of CX43, $C_{ex}$, as a percentage of tissue area for images *a*, *b* and *c*, and corresponding values for the noise level *r*. Ratios of $C_{ex}$ in images *a* and *b* with respect to image *c* are presented in the last two columns.

${\mathrm{thr}}_{c4}$	$C_{\mathrm{ex},a}$(%)	$r_{a}$(%)	$C_{\mathrm{ex},b}$(%)	$r_{b}$(%)	$C_{\mathrm{ex},c}$(%)	$r_{c}$(%)	$C_{\mathrm{ex},a}/C_{\mathrm{ex},c}$	$C_{\mathrm{ex},b}/C_{\mathrm{ex},c}$
252	1.41	0.111	1.53	0.063	2.63	0.353	0.54	0.58
253	0.82	0.039	0.97	0.031	1.60	0.169	0.51	0.61
254	0.27	0.007	0.31	0.007	0.58	0.039	0.47	0.53

**Table 3:** Morphological measurements (*L*, *W*, *R* and *A*) and $C_{lat}$ values for automatically detected CMs in images d1, d2 and *e* under the supervised mode. $d1_{CM,i}$, $d2_{CM,i}$ and $e_{CM,i}$, i∈{ 1, 2, 3, 4}, denote the evaluated CMs in images d1, d2 and *e*, respectively, which are shown in Figures 14 and 16.

CM	*L* (μm)	*W* (μm)	*R*	*A* (μm${}^{2}$)	$C_{\mathrm{lat}}$ (%)
$d1_{CM,1}$	166.9	44.5	3.7	7428.6	48.8
$d1_{CM,2}$	120.3	25.2	4.8	3031.7	60.2
$d2_{CM,3}$	107.7	35.1	3.1	3777.3	5.4
$d2_{CM,4}$	108.2	25.2	4.3	2730.6	0.0
$e_{CM,1}$	81.6	35.6	2.3	2903.8	44.1
$e_{CM,2}$	111.1	33.3	3.3	3695.3	41.6
$e_{CM,3}$	42.7	17.9	2.4	762.9	0.0
$e_{CM,4}$	71.1	33.8	2.1	2399.0	11.9

**Table 4:** Processing time for manual and automatic methods. $T_{a}$ is the time taken by the automatic method to generate the CM mask in a single core computer. $T_{at}$ is the total processing time including CM mask generation, box enclosing, box partition, quantification of morphological CM measures, CX43 characterization and statistical analysis. $N_{a}$ and $N_{m}$ denote the number of CMs in the automatic and manual masks, respectively. *S* is the summed size of the three channels in the image. $N_{a}/T_{a}$ and $N_{m}/T_{m}$ are the average number of CMs delineated per s by the automatic method and by a human expert in the field using advanced graphics tools, respectively. In brackets, the results obtained for rat images with the automatic method working in supervised mode are presented.

ID	$N_{m}/T_{m}$	$T_{a}$ (s)	$N_{a}/T_{a}$	Ratio	$T_{\mathrm{at}}$ (s)	$N_{a}$	*S* (Mb)
a	0.11	1.0	66.0	600	22	66	52.0
b	0.11	15.4	29.4	267	1036	453	363.0
c	0.11	3.4	18.8	171	54	64	92.5
d1	0.11	4.6	24.1 (6.1)	219 (55)	201 (522)	111 (28)	18.3
d2	0.11	4.6	36.9 (9.6)	335 (87)	300 (683)	170 (44)	43.5
e	0.11	2.1	7.1 (3.3)	64 (30)	26 (71)	15 (7)	18.7

## Supplementary Materials

The following are available online at https://www.mdpi.com/2218-273X/10/9/1334/s1.

## Abbreviations

The following abbreviations are used in this manuscript:

CX43	Connexin43
CM	Cardiomyocite
WGA	Wheat Germ Agglutinin
LV	Left Ventricle
SL	Sarcomere Length
SERCA	Sarco/endoplasmic reticulum $Ca^{2+}$ ATPase
DsRed	Red Fluorescent Protein
AUC	Area Under The Curve
FITC	Fluorescein isothiocyanate
