# Supplementary material for: Automatic Quantification of Cardiomyocyte Dimensions and Connexin 43 Lateralization in Fluorescence Images
Source: Biomolecules. 2020 Sep 17;10(9):1334. doi: 10.3390/biom10091334 (PMC7565961; doi:10.3390/biom10091334)
Supplement: Supplementary file 1 [file biomolecules-10-01334-s001.pdf]

biomolecules

MDPI

Supplementary file: Step-by-step guide to run MARTA

Automatic quantification of cardiomyocyte dimensions and Connexin 43 lateralization in fluorescence images

Antoni Oliver[1], Laura García-Mendívil[1], José María Vallejo-Gil[2], Pedro CarlosFresneda-Roldán[2], Katarína Andelová[4], Javier Fañanás-Mastral[2], Manuel Vázquez-Sancho[2], Marta Matamala-Adell[2], Fernando Sorribas-Berjón[2], Carlos Ballester-Cuenca[2], NarcisaTribulova[4], Laura Ordovás[1,3], Emiliano Raúl Diez[5,‡*], and Esther Pueyo[1,6, ‡*]

1 Biomedical Signal Interpretation and Computational Simulation (BSICoS), Institute of Engineering Research(I3A), University of Zaragoza, Instituto de Investigación Sanitaria (IIS), Zaragoza, Aragon, 50018, Spain

2 Department of Cardiovascular Surgery, University Hospital Miguel Servet, Zaragoza, Aragon, 50018, Spain

3 Aragon Agency for Research and Development (ARAID), Zaragoza, Aragon, 50018, Spain

4 Centre of Experimental Medicine, SAS, 84104, Bratislava, Slovakia

5 Institute of Experimental Medicine and Biology of Cuyo (IMBECU), CONICET, Mendoza, 855, 5500, Argentina

6 Biomedical Research Networking Center in Bioengineering, Biomaterials and Nanomedicine (CIBER-BBN), Zaragoza, Aragon, 50018, Spain

*Correspondence: epueyo@unizar.es; Tel.: +34-976-762963 (E.P.); diez.emiliano@fcm.uncu.edu.ar; Tel.:+54-0261-413-5000 int 2763 (E.D.)

‡These authors contributed equally to this work.

Introduction

MARTA software is designed to process images of cardiac tissue obtained by confocal fluorescence microscopy. The objective of this software is to measure morphological characteristics of cardiomyocytes (CM) and to estimate the amount of CX43 as well as its lateralized proportion from longitudinal cardiac tissue sections. To do this, the software can process a single merged image or multiple separate images (each corresponding to an independent marker).

The steps to run the developed software in 64-bit Windows OS are provided below in video format.

Video Demonstration

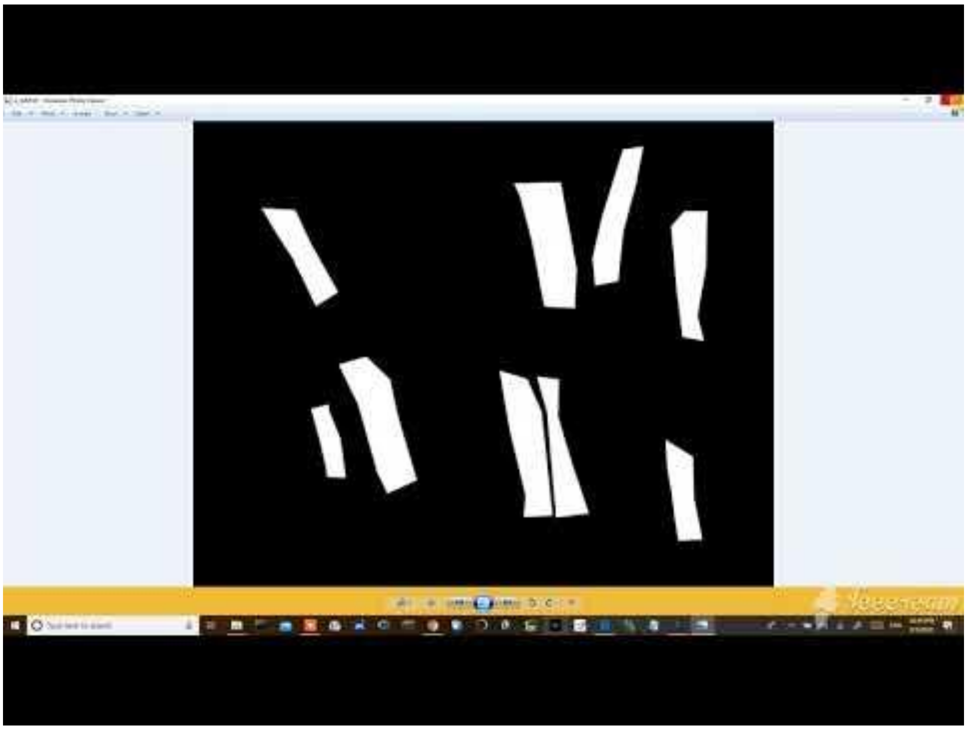

Step-by-step explanation

1. Download the program available in the GitHub repository. Select code and Download ZIP (https://github.com/tonibois/MARTA):

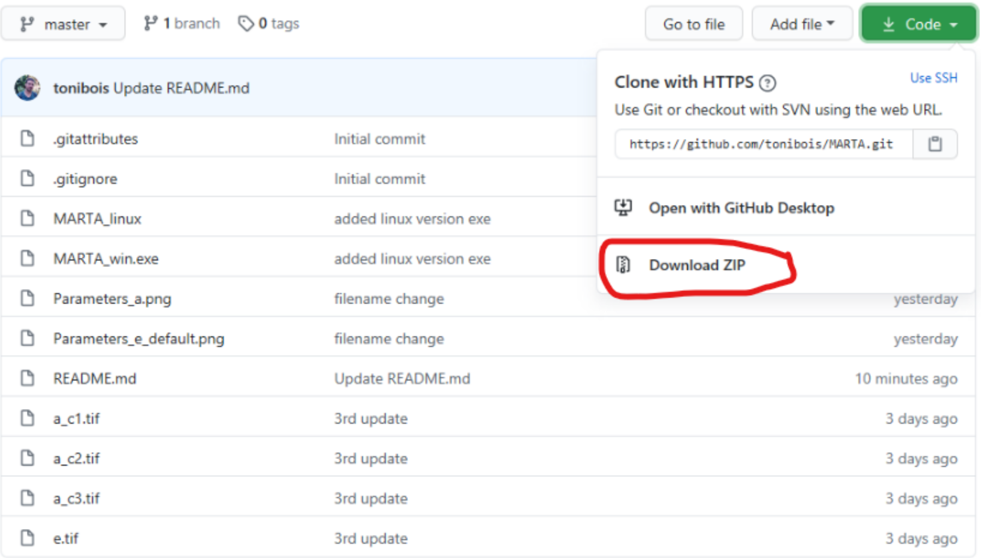

2. Once the ZIP has been downloaded, unzip it into a folder:

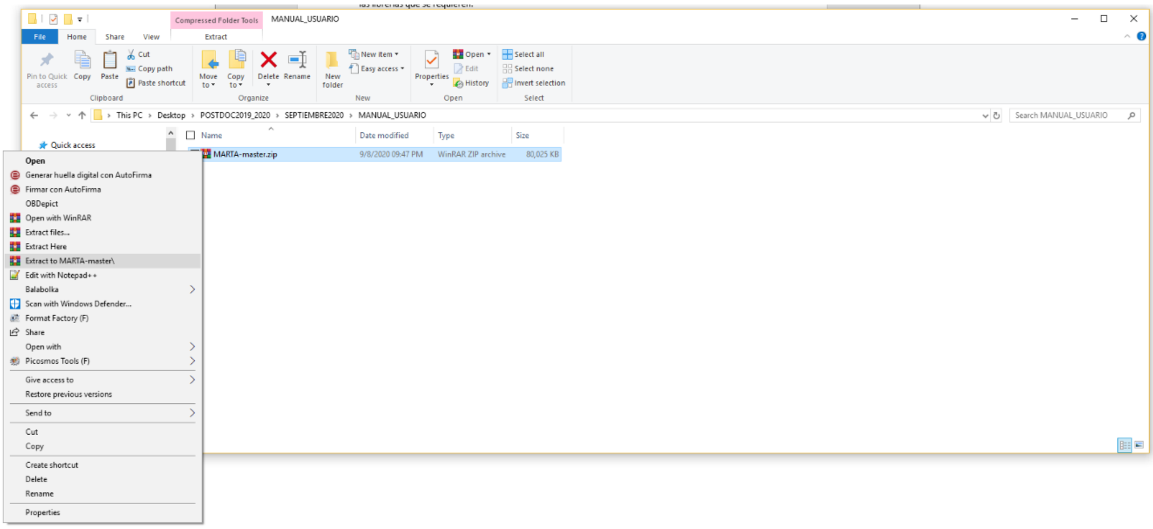

3. Navigate to find the executable file MARTA_v3.exe in the unzipped folder:

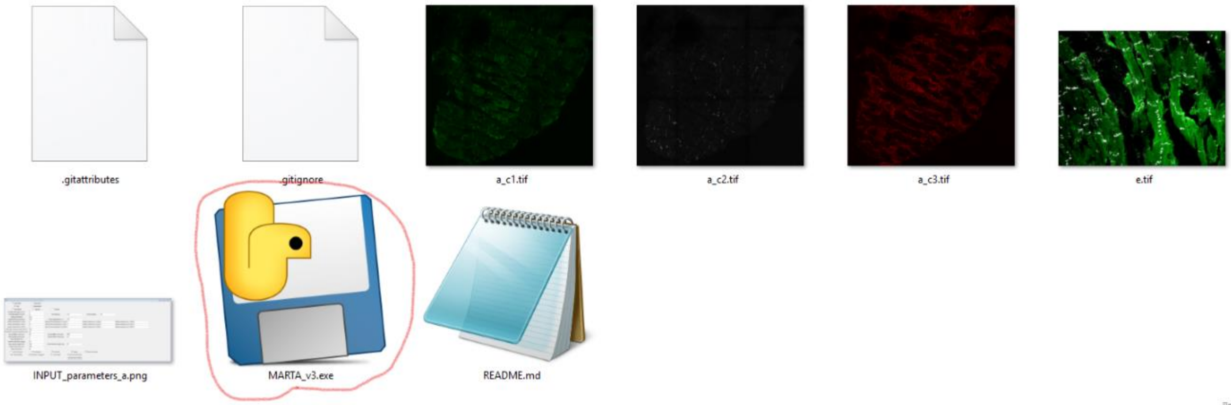

To process images, it is important that the images to be processed are located in the same directory as the executable file MARTA_v3.exe. Two test cases have been included in the repository, which correspond to images a and e:

- a
  - a_c1.tif (SERCA)
  - a_c2.tif (CX43)
  - a_c3.tif (WGA)
- e
  - e.tif (F-Actin + Cx43)

4. Double click on the icon corresponding to the executable MARTA_v3.exe. The following screen will appear (default settings configured to process the image "e.tif")

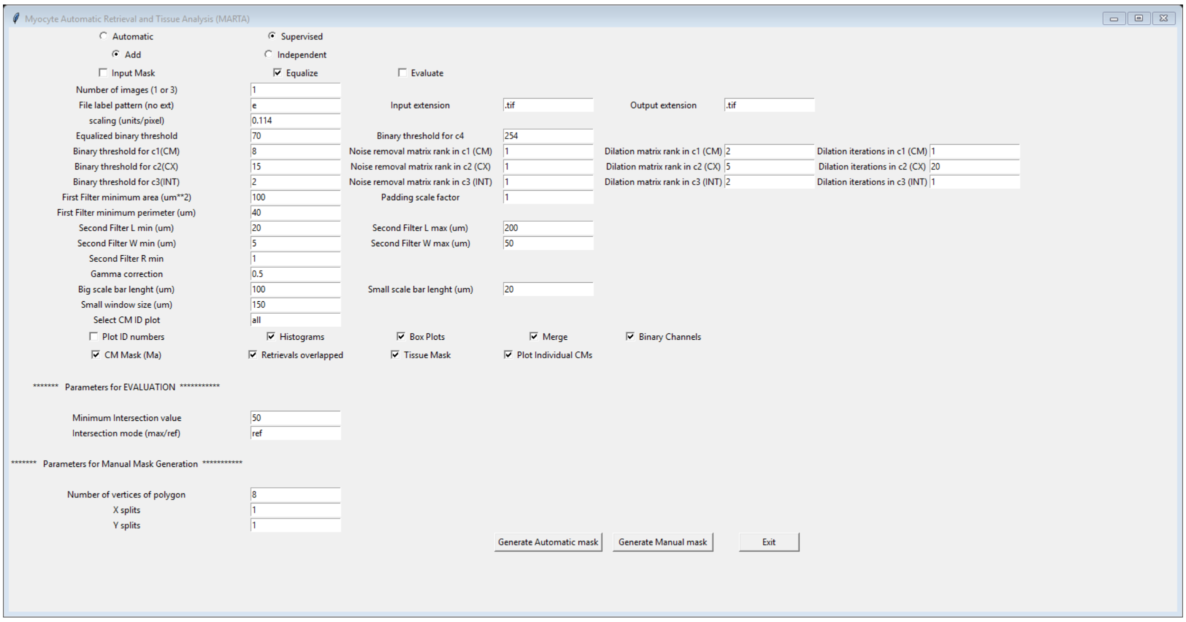

To help in filling in the values for each of the parameters used by the software, hovering over a blank field provides additional information about the parameter contained in that field:

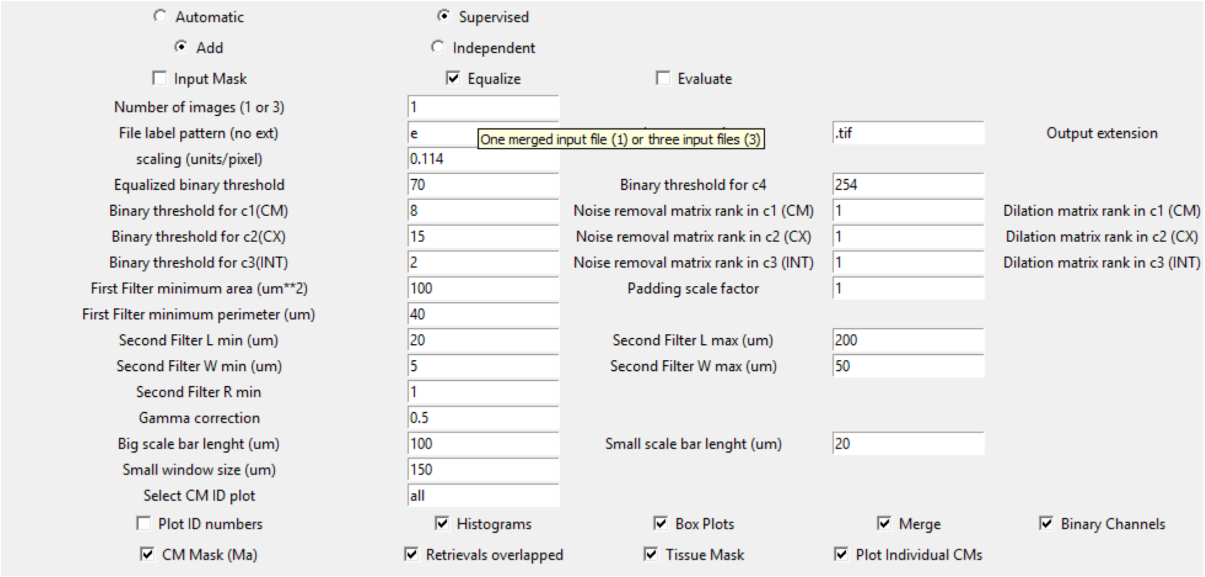

AUTOMATIC ANALYSIS OF SINGLE MERGED IMAGE (e)

To process an image in automatic mode using the default settings, select the *Automatic* checkbox:

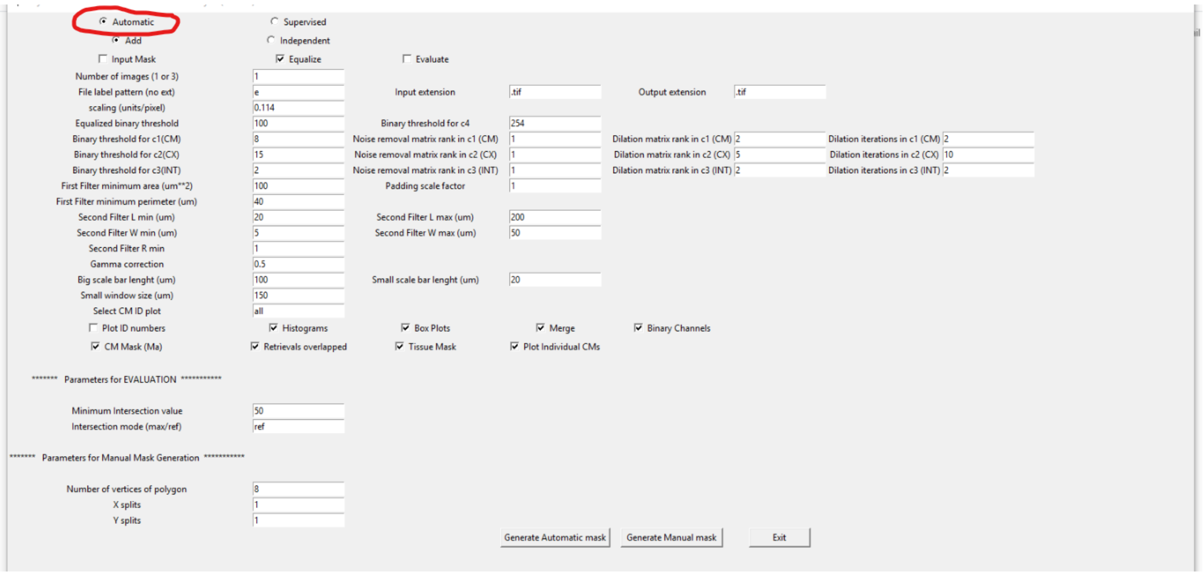

Next, press the *Generate Automatic mask* button:

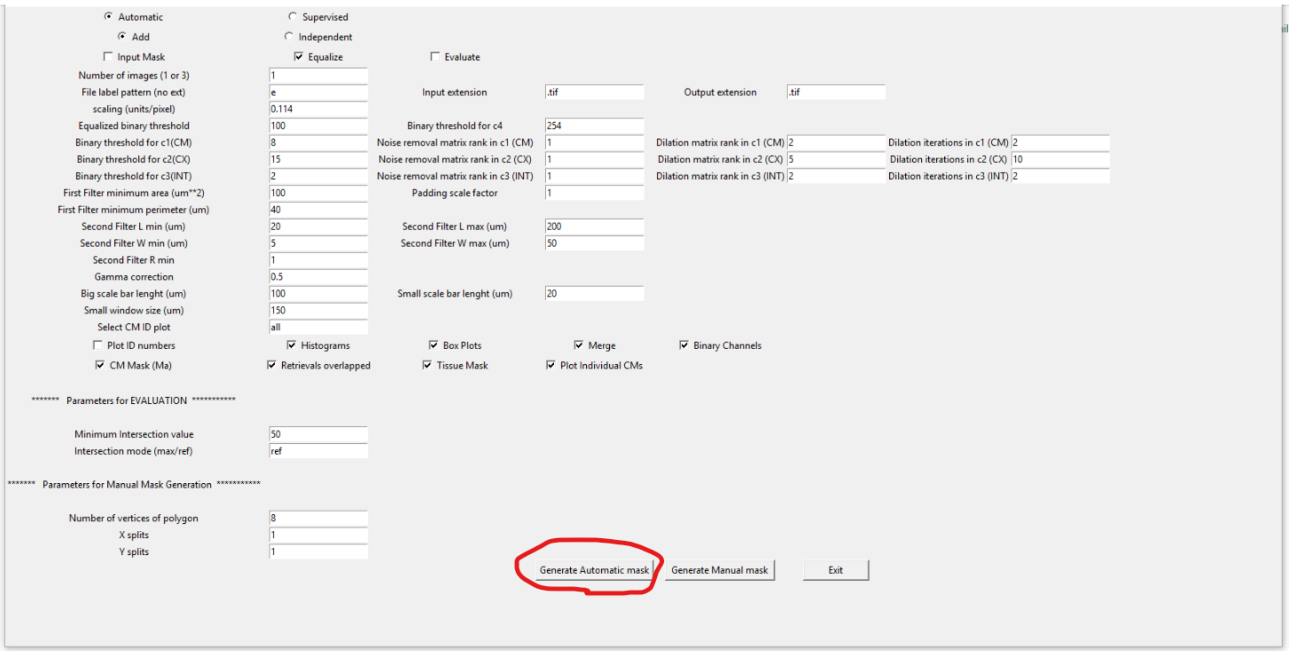

In a few seconds (about 16 seconds on an i7 processor), the mask output by the software is generated in a folder created within the same directory. Also, the artificial separation of channels c1 (CM), c2 (CX43) and c3 (Interstitial) corresponding to the e_c1.tif, e_c2.tif, and e_c3.tif images is obtained.

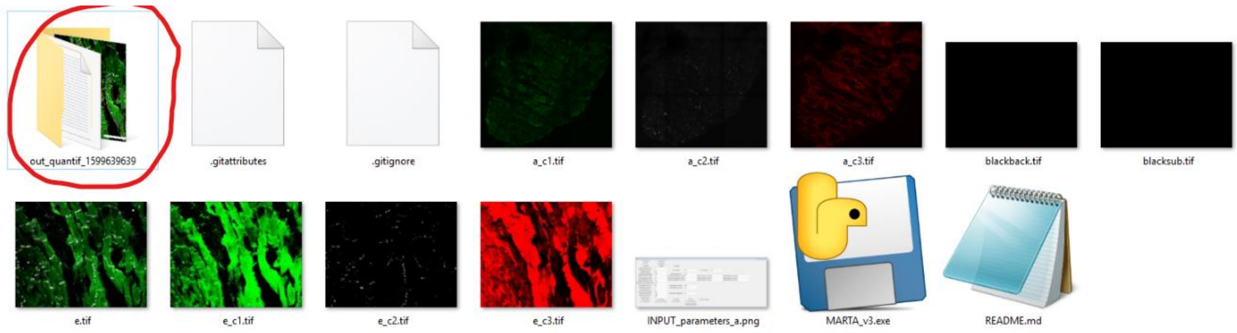

By navigating in this folder generated by the software, the results of the processing in the form of files containing information about each individually detected CM as well as global statistical information from all CM detections can be found.

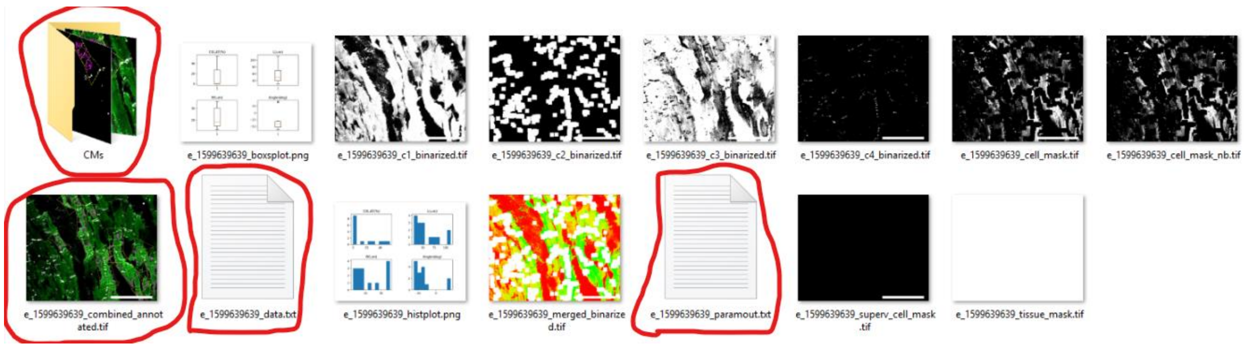

To get the statistical summary, go to the end of the generated "e_X_paramout.txt" file (where X is a time-dependent character string):

```
********************************************************************************************
******************************  CHANNEL PROPERTIES  ****************************************
********************************************************************************************
RGB mean of input:                                33.28
********************************************************************************************
***********************  RELATIVE PROPORTIONS OF BIOMARKERS  *******************************
********************************************************************************************
Percentage of c2 respect to c3 and c1 (CXEX in %):   0.7
Percentage of c3 respect to c3 and c1 (INEX in %):   36.41
Percentage of c1 respect to c3 and c1 (CMEX in %):   62.89
********************************************************************************************
********************************************************************************************
Intersection between c1 and c3 (%):                 38.64
Tissue area from pixel count (mm2) :                 0.07936
***********************  RESULTS STATISTICAL ANALYSIS  *************************************
Number of detected CMs:                              15
Percentage of Cx43 Lateral (%):                      14.17+/-18.82
Average Lenght of CMs found (um):                    57.83+/-24.58
Average Width of CMs (um):                           21.61+/-8.64
Average angle of CMs (degree):                       -33.01+/-30.4
***********************  RESULTS COMPUTING TIME  *******************************************
Total time of execution (s) :                        20.64
********************************************************************************************
```

As an example of the retrieved statistical information when processing image e, , the percentage of lateral CX43 is shown to be 14.7% for a number of detections equal to 15, with an average length of about 58 microns and an average width of 21.6 microns. For information of each CM detection, the user is referred to the *e_X_data.txt* file. This file can be read as a comma separated file (CSV) using, for instance, *Microsoft Excel* or *LibreOffice.*

In addition, individual CM detections can be appreciated in the same folder generated by the software:

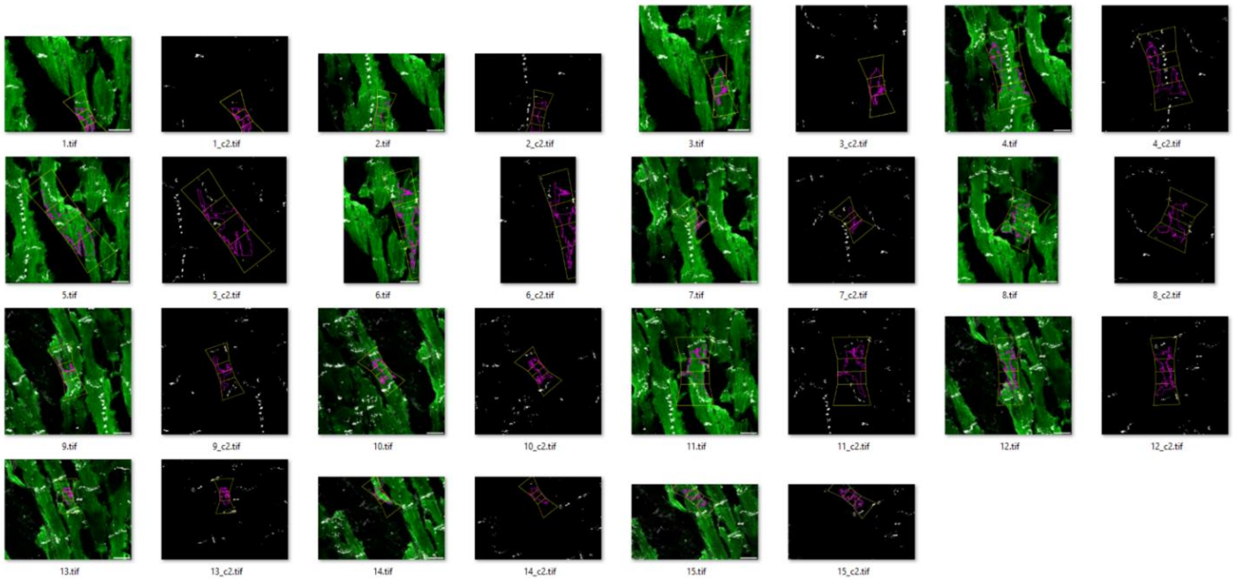

AUTOMATIC SUPERVISED ANALYSIS OF SINGLE MERGED IMAGE (e)

The supervised option for this same image "e" is selected by default when the program is run. In any case, it can also be selected in the first row of options at the top right of the screen under the name *Supervised*.

The “*Generate Automatic mask*” button is then pressed again when the *Supervised* option is selected. After doing this, the first detected CM is obtained. The user is asked to decide if he/she wants to save the detection by entering "y" in the dialog. If he/she decides to save the detection accepting that it is a CM, the following will be seen in the screen:

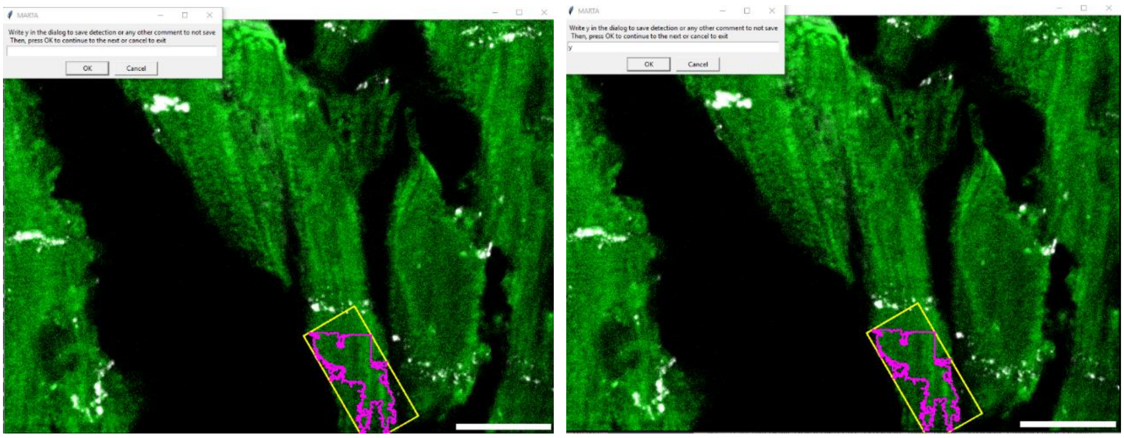

Once "y" has been entered in the dialog window, press OK to continue with the next detection. Only the selected CMs with “y” will be displayed in the final image. However, the data file (*e_X_data.txt*) will still include all CMs with the introduced text assigned to each detection (column "value"). Only CMs saved with the “y” value are displayed in the final image (*X_combined_annotated.tif* CMs). The CMs folder additionally displays all the detected CMs with the entered “value” being part of their filenames:

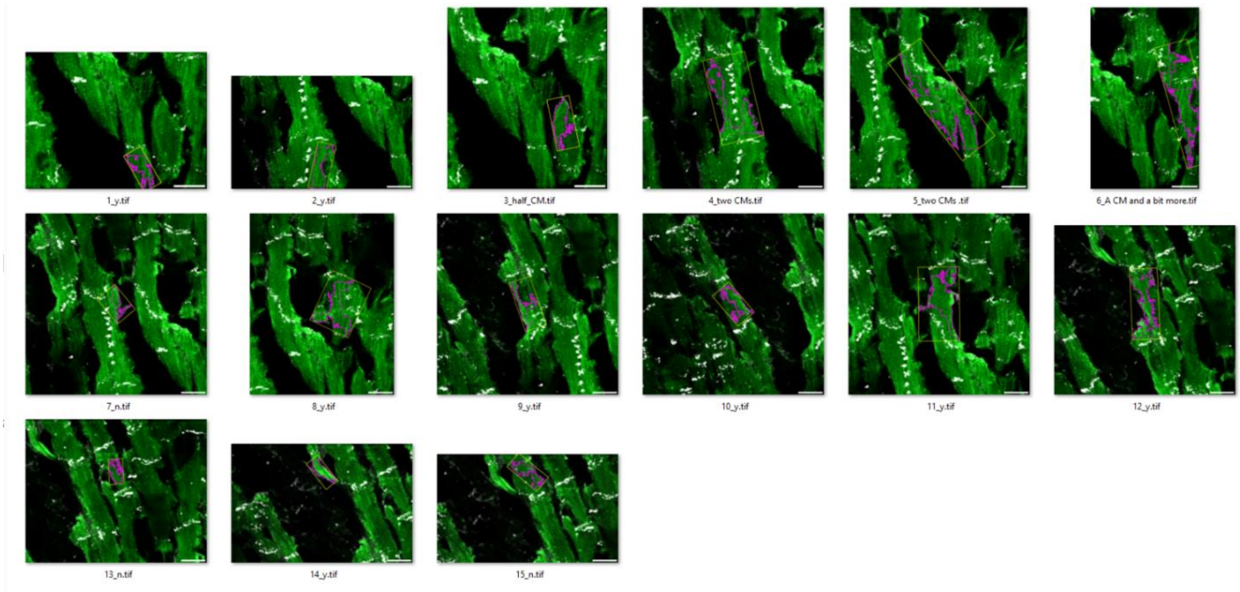

MANUAL MASK GENERATION FOR SINGLE MERGED IMAGE (e)

To assess the performance of the automatic method (in supervised or non-supervised mode), a reference mask for the original image "e" is required. The automatic and reference masks should have the same number of pixels and the same dimension. The reference mask can be generated with an external program like GIMP or KRITA’s and introduced as an input to the software by enabling the *Input Mask* option. Also, there is the possibility to generate it using MARTA software by clicking the *Manual Mask Generation* button.

The name of the file containing the reference for the image named “e” should be *e_mM* and this should be in the format chosen in the parameter row (common formats are TIFF, PNG and JPG). By default, the input and output images will be in tif format. Therefore, if a manual mask is input, it should be called *e_mM.tif* for this case. The software will already provide this name to the manual output mask.

In the following, the generation of a manual mask using MARTA software is illustrated. First, select the values of the parameters in the bottom part of the screen (see next figure):

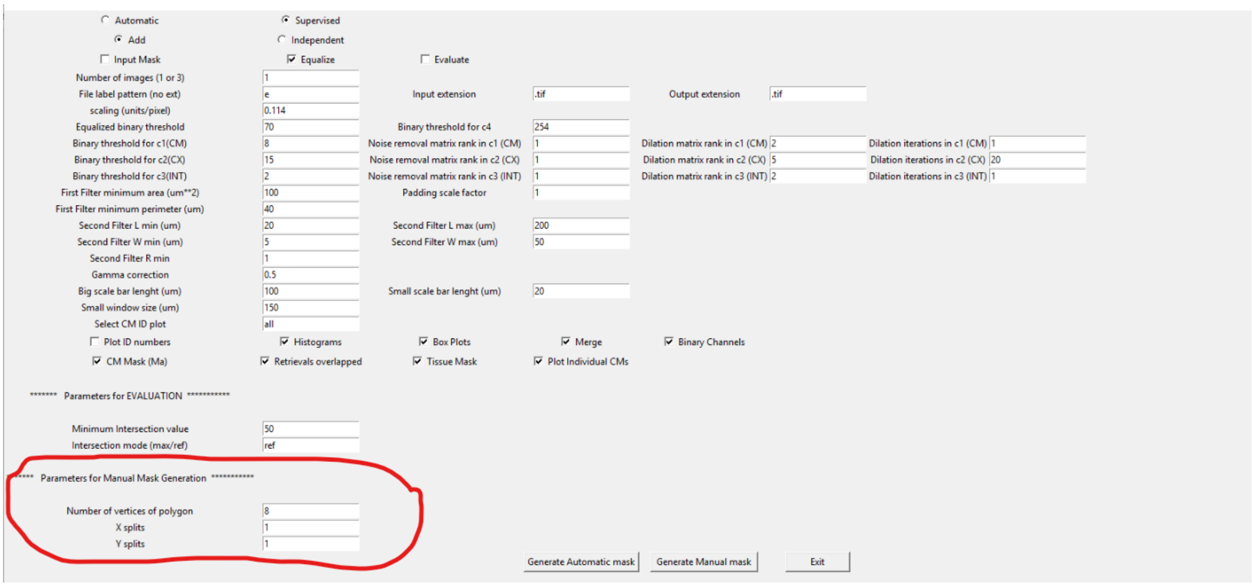

By default, an 8-vertex polygon is set to draw the boundary of each CM, but the number of vertices can be any natural number. In this example, the default value of 8 is set. Next, the user should decide in how many parts he/she would like to split the original image to contour each part sequentially. Let's choose values of 3 and 2 for X splits and Y splits, respectively, rather than values of 1 and 1 as in the default case. Next, press the *Manual Mask Generation* button in the screen (with X splits = 3, Y splits = 2 and Number of vertices of the polygon = 8).

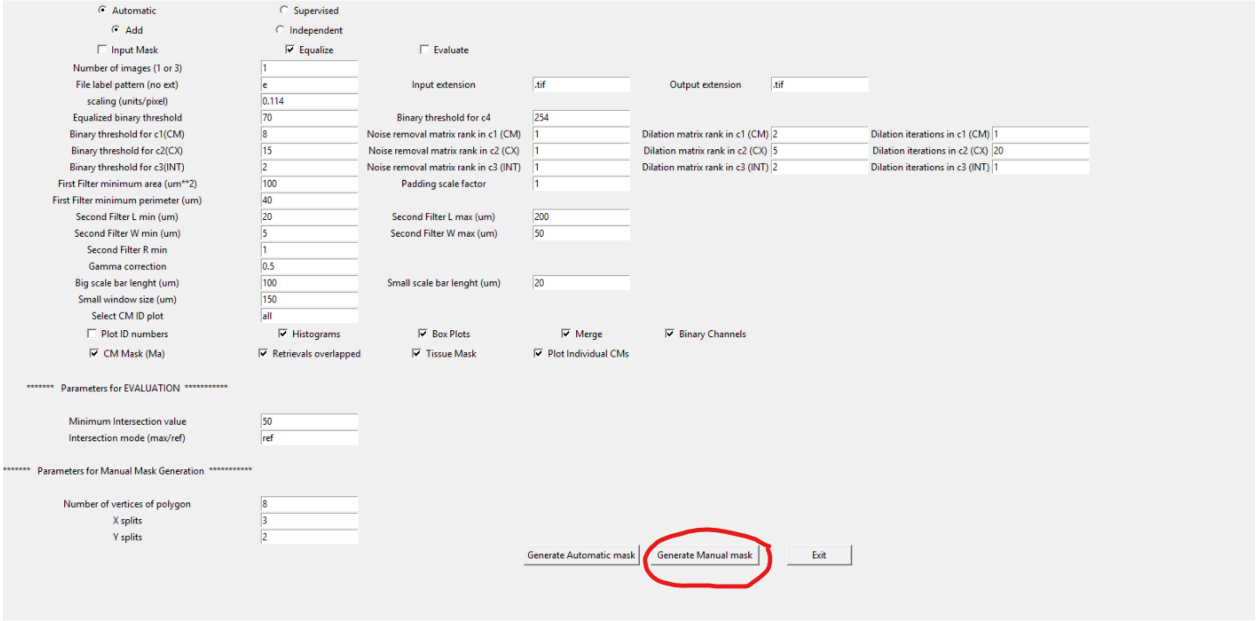

A pop-up window will appear, in which mouse **double-clicking** allows setting the vertices of the polygon (which are numbered to help following the line connections). When one CM delineation is finished, the user can move to the next CM by pressing ESC.

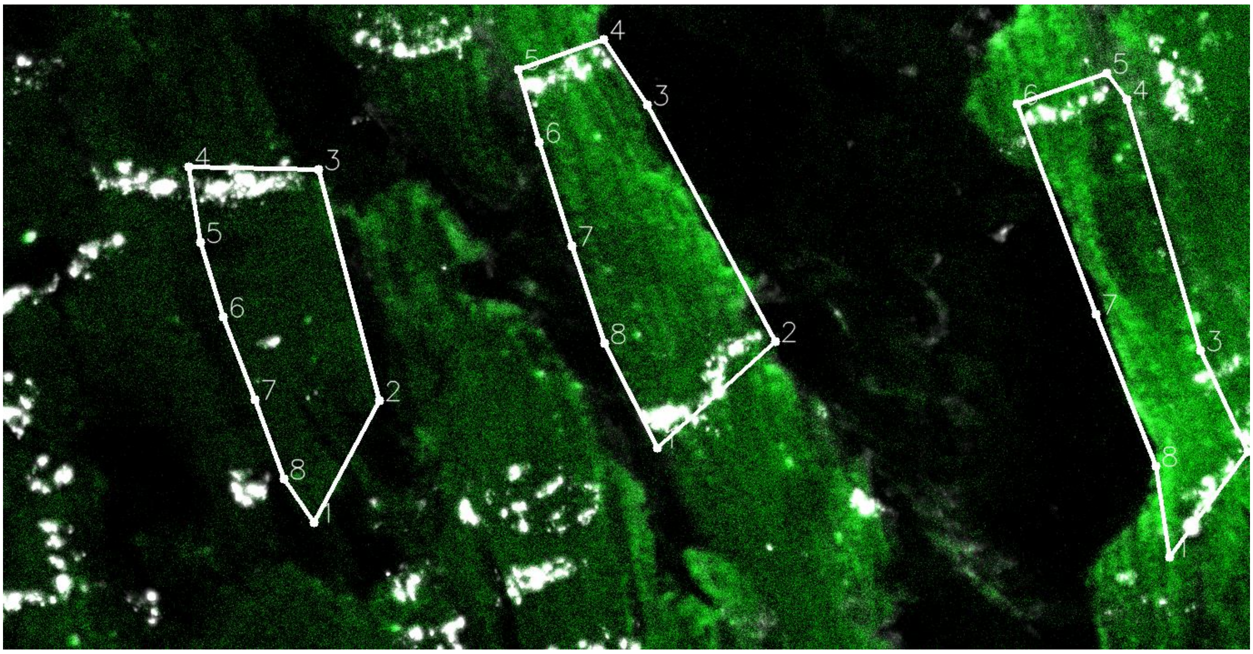

At the end of the manual delineation, the image and the generated mask are presented:

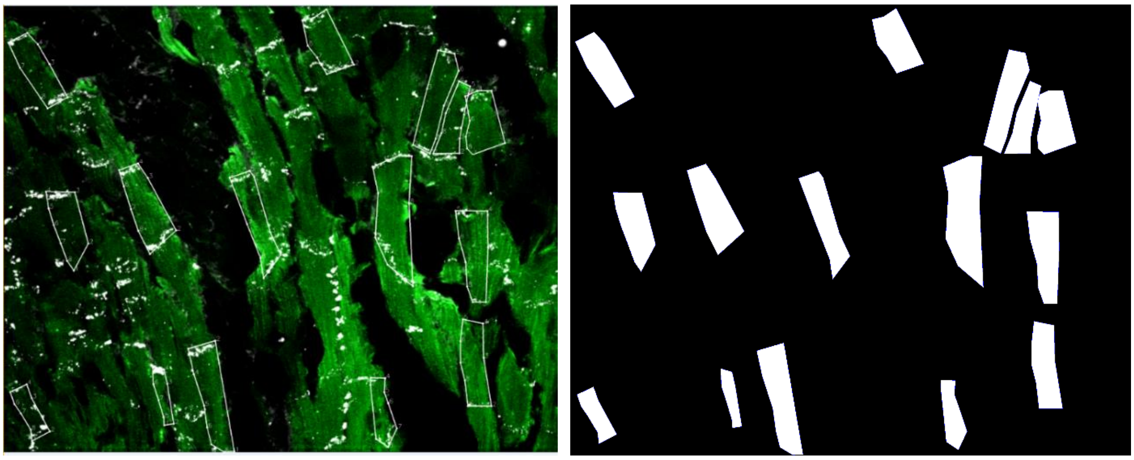

If the user selects *Input Mask,* the software will go on to parse the generated image *"e_mM.tif"* within the directory where the executable is located (not within any subfolder). If *"Input Mask"* is deselected and the *Evaluate* option is selected, the software will evaluate the correspondence between the automatic mask and the manual mask when the *"Generate Automatic Mask"* button is pressed. These results will appear in the newly generated subfolder.

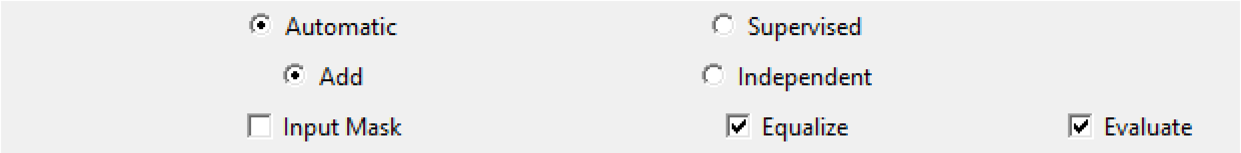

In addition, the user can configure associated parameters like the minimum percentage of match required between the two masks and the evaluation method (i.e. intersection area

between the two masks divided by the reference area (*ref*) or by the maximum area between reference (*manual*) and test (*automatic*), which is called “max” method. If a label different from *max* or *ref* is specified, then *ref* method will be used.

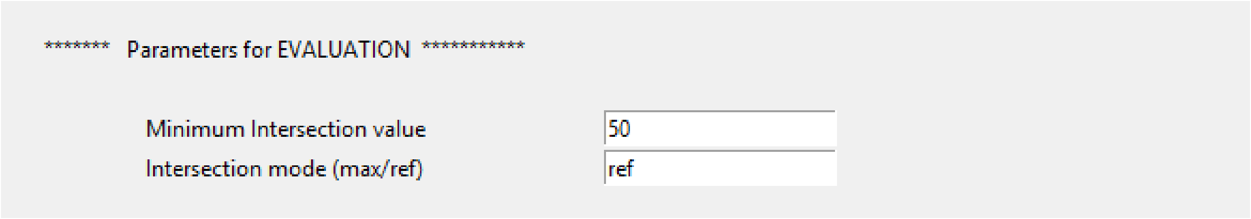
******* Parameters for EVALUATION ***********

Minimum Intersection value 50
Intersection mode (max/ref) ref

Once the execution has finished, press the *Exit* button to exit the program and view the written files:

File	Description
e_X_autocoinc_data.txt	Individual CM info for CMs of the automatic mask presenting high intersection with manually detected CMs
e_X_evaluation_output.txt	Evaluation summary
e_X_manucoinc_data.txt	Individual CM info for CMs of the manual mask presenting high intersection with automatically detected CMs
e_X_MaxAnd.tif	CMs with high coincidence between manual and automatic masks
e_X_Mm_Ma_cont.tif	Overlap between manual and automatic masks

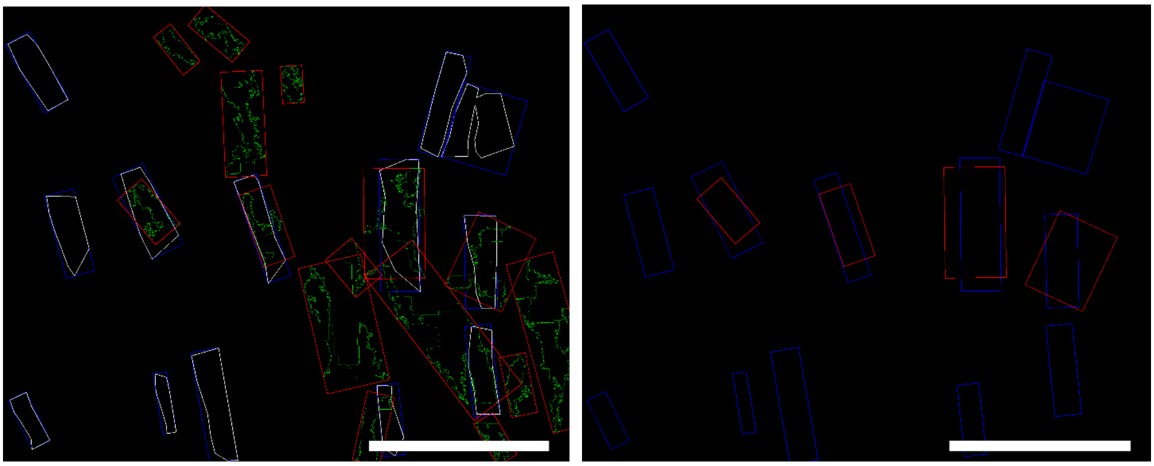

AUTOMATIC ANALYSIS OF THREE-CHANNEL IMAGE (a)

To perform the same analysis with an image with three separated channels, those should be named under the same identifier, for example "a", which should be entered in the graphical interface in the field "*File label pattern*". In addition, specify that there are 3 images in Number *of images* (1 or 3). In addition, the values of the parameters to process that image,

such as the binarization threshold and parameters related to noise reduction and dilation, should be adjusted. In the following example, the image is not equalized and the parameter values are set out as in Table 1 of the main manuscript. If there is no equalization, the parameters regulating binarization are those marked in red in the following image:

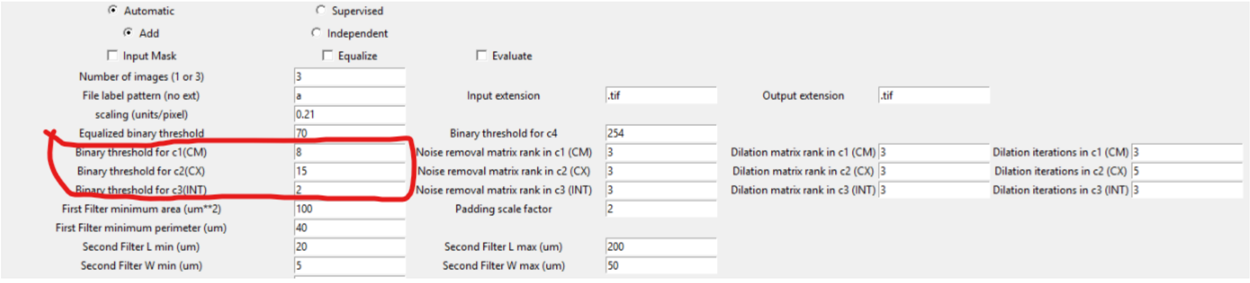

The values of the parameters related to erosion and dilation for noise removal are set in the fields on the right part of the screen. In the bottom part of the screen, values for the parameters related to area, perimeter, length and width filters are set.

With the following parameter values, the software can be run by pressing the *"Generate Automatic Mask"* button:

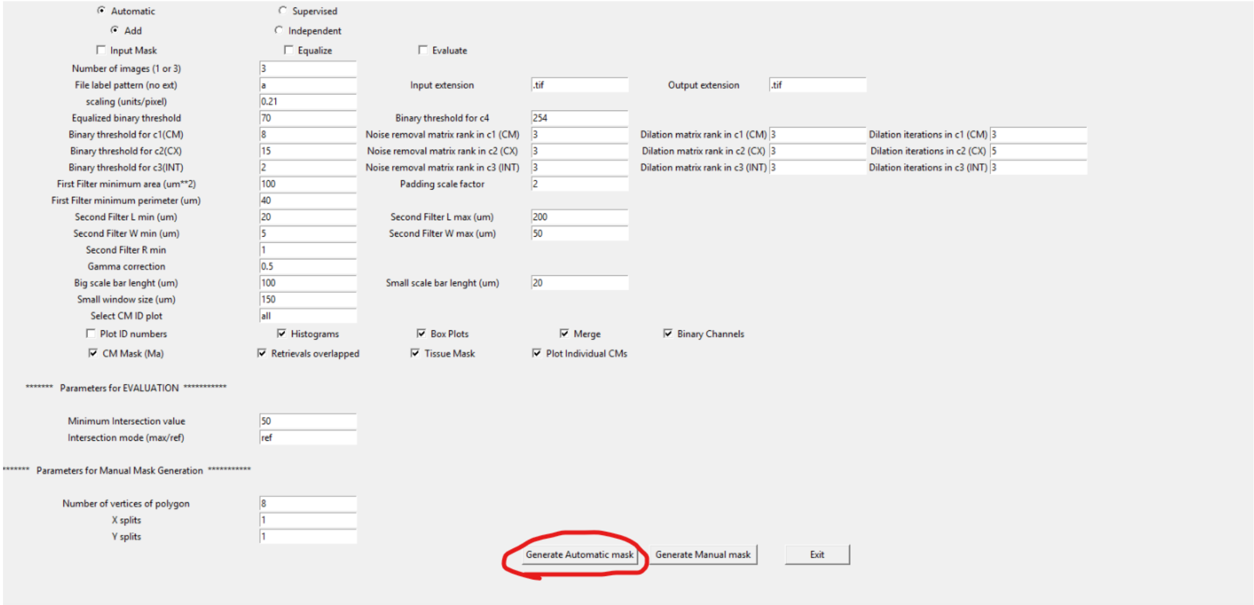

The software will then generate an "*out_quantifout_quantif_X*" folder with the processing results. To access this material in video form, the user is referred to the online tutorial.
